# Supplementary material for: Guest-adaptive molecular sensing in a dynamic 3D covalent organic framework
Source: Nat Commun. 2022 Dec 24;13:7936. doi: 10.1038/s41467-022-35674-8 (PMC9790004; doi:10.1038/s41467-022-35674-8)
Supplement: Supplementary file 1 — Supplementary Information [file 41467_2022_35674_MOESM1_ESM.pdf]

Supplementary Information (81 pages)

Guest-adaptive molecular sensing in a dynamic 3D covalent organic framework

Lei Wei,[1,#] Tu Sun,[1,2,#] Zhaolin Shi,[1] Zezhao Xu,[1] Wen Wen,[3] Shan Jiang,[1] Yingbo Zhao,*[,1,2] Yanhang Ma,*[,1,2] and Yue-Biao Zhang*[,1,2]

[1]*School of Physical Science and Technology, ShanghaiTech University, Shanghai, 201210, P. R. China.*

[2]*Shanghai Key Laboratory of High-Resolution Electron Microscopy, ShanghaiTech University, Shanghai 201210, China.*

[3]*Shanghai Synchrotron Radiation Facility, Shanghai Advanced Research Institute, Chinese Academic of Sciences, Shanghai 201210, China.*

*To whom correspondence should be addressed: zhaoyb2@shanghaitech.edu.cn; mayh2@shanghaitech.edu.cn; or zhangyb@shanghaitech.edu.cn

[#]These authors contributed equally.

Table of Contents

Supplementary Section 1. Material preparation and characterization 3

Supplementary Section 2. 3D electron diffraction & structural modeling 18

Supplementary Section 3. Synchrotron powder X-ray diffraction analyses 26

Supplementary Section 4. Fluorescence sensing of organic gas/vapours. 44

Supplementary Section 5. *In-situ* fluorescence spectroscopy for dynamic multi-component gas sensing. 57

Supplementary Section 6. Computational simulation 63

Supplementary Section 7. Tables of fractional atomic coordiantes 64

Supplementary References 81

Section 1. Material preparation and characterization

Instrumentation. The scanning electron microscopy (SEM) images of COF samples were collected on the JSM-7800F PRIME extreme-resolution field emission SEM. The sample was suspended in 1,4-dioxane and dip-coated on the silicon sample holder for characterization under high vacuum. The working distance and voltage were optimized for the visualization of the crystals.

Single crystals were mounted on a Bruker D8 Venture diffractometer equipped with a fine-focus Cu target X-ray tube operated at 40 W power (50 kV, 1 mA). The specimen was cooled to 150 K using an Oxford Cryosystem chilled by liquid nitrogen. Bruker APEX2 software package was used for data collection; SAINT software package was used for data reduction; SADABS was used for absorption correction; no correction was made for extinction or decay.

The Fourier-transform infrared (FT-IR) spectra were recorded on neat samples in the range of 500–4000 cm^{-1} on a PerkinElmer FT-IR spectrometer equipped with single reflection diamond ATR module.

The solid-state ^{13}C nuclear magnetic resonance (^{13}C SSNMR) spectra were recorded on a Bruker ADVANCE 400 MHz Solid NMR spectrometer with cross-polarization magic angle-spinning (CP/MAS) and 3.2-mm double-resonance MAS probe.

The samples were loaded in the zirconia rotor with a sample spinning rate of 13.0 kHz. The thermal gravimetric analyses (TGA) were carried out using a TAQ50 TGA analyzer from 25 to 800 °C under N_2 atmosphere with a temperature ramping rate of 5 °C/min.

Low-pressure gas adsorption isotherm was measured volumetrically using a Quantachrome iQ (CO_2). Dry ice-methanol bath was used for temperature-controlled at 195 K. Water bath with circulator was used for temperatures controlled at 298 K.

Synthesis of AnDA[1]: A solution of veratrole (40 mmol, 1 equiv, 5.36 g.) and the terephthalaldehyde (TPA, 40 mmol, 1equiv, 5.364 g.) in dichloromethane (30 mL) was added to dropwise to 84% sulfuric acid (40 mL) while the temperature was kept between 0 and 5°C. After the addition, the suspension was stirred for 2.0 h at room temperature. The reaction was quenched with water and neutralized by ammonia. After extraction with dichloromethane, the solvent was stripped off in vacuo. Then the crude product was purified chromatographically with $CHCl_3$ and ethyl acetate, affording AnDA in 40% yield; ^{1}H NMR (500 MHz, CDCl3) δ 10.24 (1H, s, CHO), 8.19 (2H, d, J = 8.0 Hz, 2-6-H phenyl), 7.71 (2H, d, J = 8.0 Hz, 3-5-H phenyl), 6.73 (2H, s, 1,8-H anthracene), 3.74 (6H, s, OCH3). ^{13}C NMR (100 MHz, $CDCl_3$, ppm) δ 192.152, 149.302, 146.696, 135.667, 132.019, 131.915, 130.249, 125.359, 103.352, 55.591.; MS (EMI) m/z 507.1785.

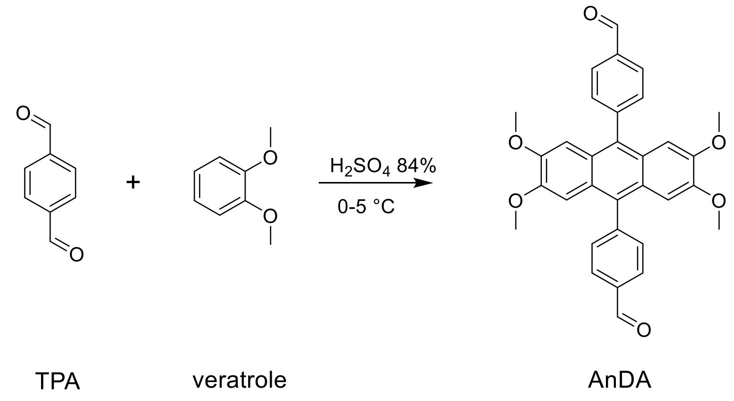

Supplementary Figure 1. The synthesis route of the starting material AnDA.

Synthesis of dynaCOF-330. AnDA (60 mg, 0.119 mmol) was dispersed in 2.5 mL of 1,4-dioxane, and then 0.5 mL of aqueous acetic acid (6 M) and 150 μL of aniline were added to a 20 mL vial. The mixture is dispensed by ultrasonication, and then the solution of TAM (25 mg, 0.066 mmol) dissolved in 2.5 mL of 1,4-dioxane was added. The reaction was heated at 80 °C for three days; the light yellow-coloured solid was separated by centrifugation, and washed with 1,4-dioxane for three times. The solid was then evacuated at 100 °C for 10 hours to obtain dynaCOF-330 (45 mg, 62% yield).

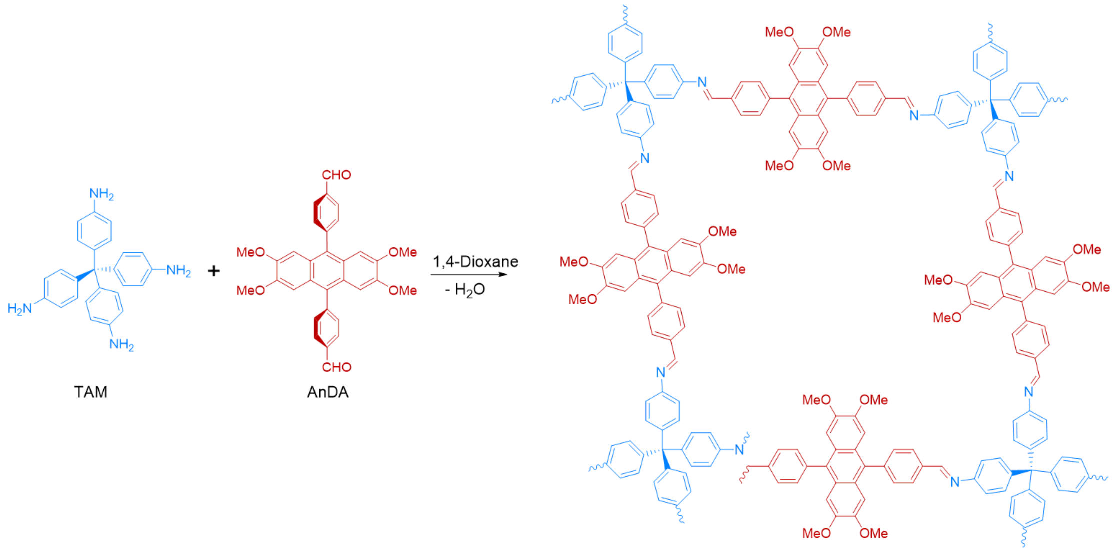

Supplementary Figure 2. The synthesis route of dynaCOF-330.

Synthesis of molecular model compound M-1 ((1E,1'E)-1,1'-((2,3,6,7-tetramethoxyanthracene-9,10-diyl)bis(4,1-phenylene))bis(N-phenylmethanimine)). A mixture of AnDA (24 mg, 0.0474 mmol) and aniline (80 μL, 0.876 mmol) in 1,4-dioxane (1 mL) were added to the 4 mL vial. The reaction was heated at 80 °C for one day and light-yellow single crystal was collected by directly cooling down the mixture at room temperature. Single crystal structure of M-1 is shown in Supplementary Figure 1. Then the solvent was stripped off in vacuo and bright yellow solid was washed several times with EtOH which was collected by filtration and dried under vacuum (31 mg, 99% yield). ^{1}H NMR (500 MHz, $CDCl_3$) δ 8.6 (s, 4H), 7.99 (d, 8H, J = 7.6 Hz), 7.72 (d, 8H, J = 8.1Hz), 7.65 (d, 8H, J = 7.6 Hz), 7.47 (t, 8H, J = 7.6 Hz), 7.39 (t, 4H, J = 7.3 Hz), 7.33 (d, 8H, J = 8.5 Hz), 7.21 (d, 8H, J = 8.1 Hz). ^{13}C NMR (100 MHz, $CDCl_3$) δ 160.21, 149.12, 143.38, 135.57, 132.50, 131.68, 129.30, 129.27, 126.22, 125.63, 129.30, 121.00, 103.66, 55.65.

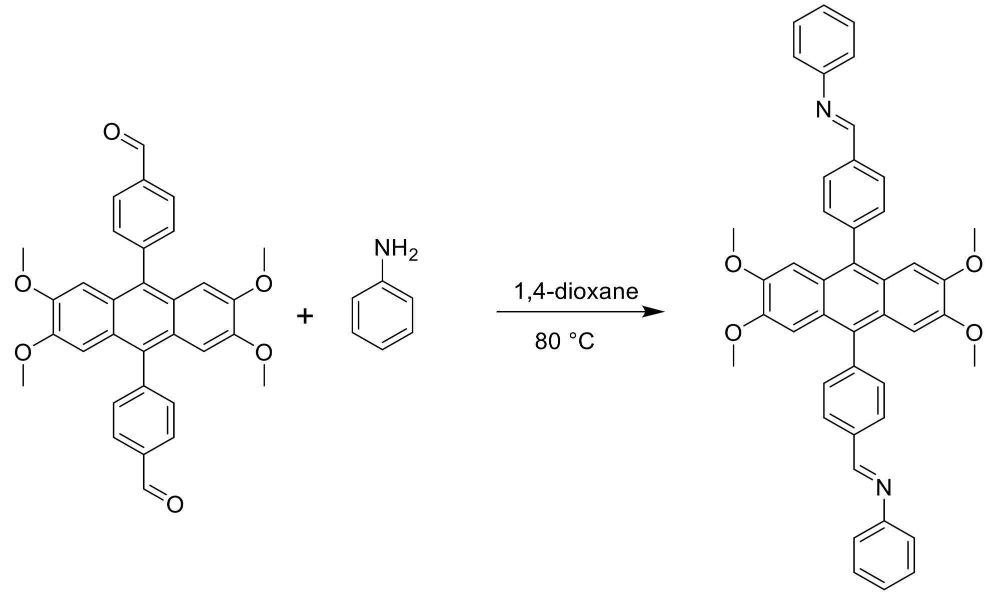

Supplementary Figure 3. The synthesis route of the molecular model compound M-1.

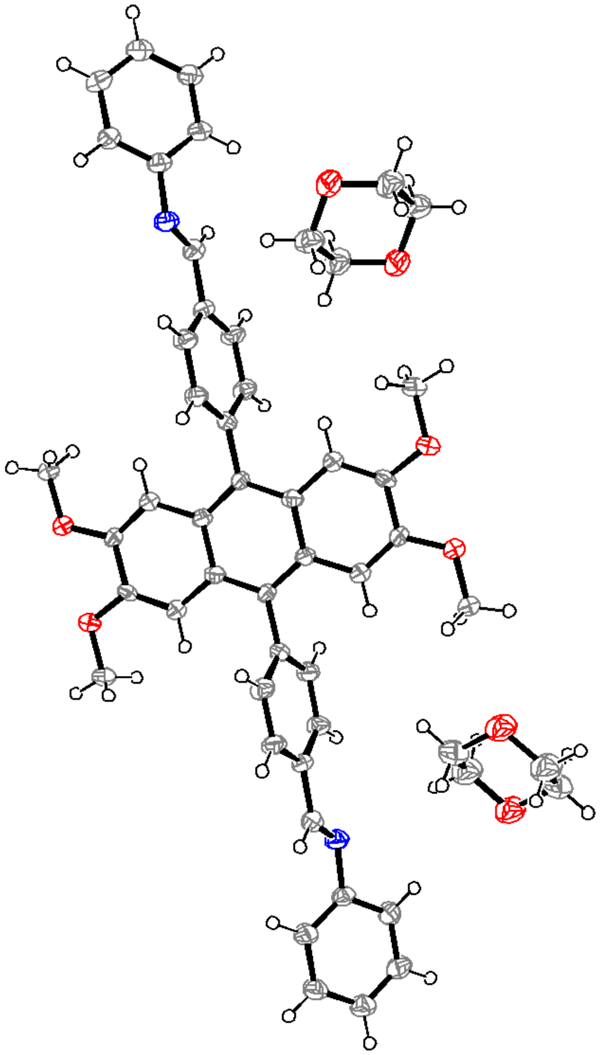

Supplementary Figure 4. ORTEP drawing of the crystal structure of **M-1**. Thermal ellipsoids are displayed with a 50% probability. Colour code: N, blue; O, red; C, grey; H, cycle.

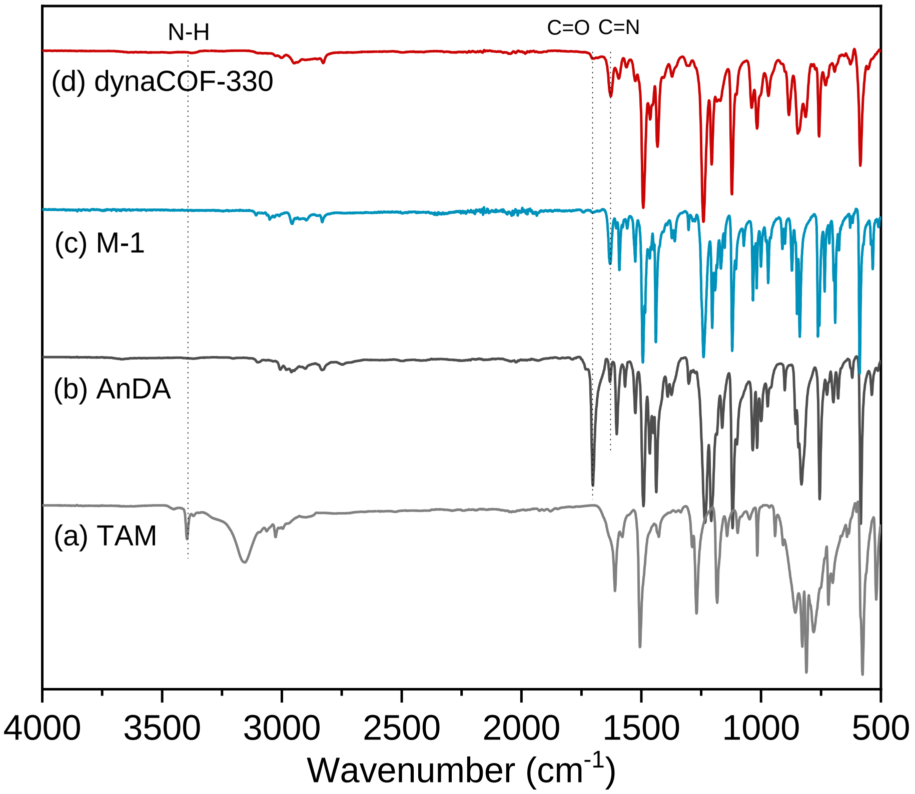

Supplementary Figure 5. FTIR spectra of starting material (TAM, AnDA), model compound M-1, and dynaCOF-330. IR peak at 3400 cm^{-1} for TAM (assigned to the N-H stretching mode of amino group) and 1704 cm^{-1} for AnDA (assigned to the C=O stretching mode of aldehyde) disappeared. Newly emerged IR peak at 1632 cm^{-1} for M-1 and 1627 cm^{-1} for dynaCOF-330 confirms the formation of imine linkage in dynaCOF-330.

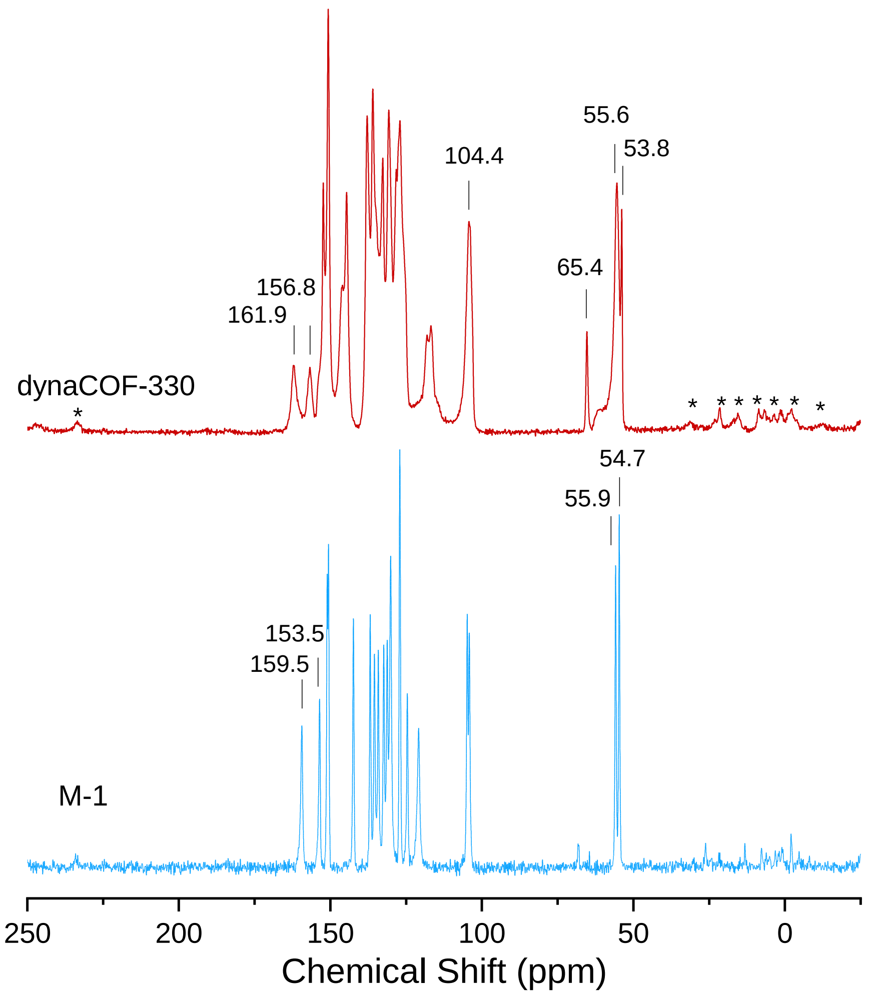

Supplementary Figure 6. The ^{13}C SSNMR spectra of dynaCOF-330 and M-1 showing the successful formation of imine linkage. The signal at 159.5 ppm was assigned to the carbon atom of imine bond for M-1. By that analogy, we assigned the signal at chemical shifts of 161.9 and 156.8 ppm of dynaCOF-330 to the carbon atom of the imine bond. The characteristic chemical shift of 55.6 and 53.8 ppm should be assigned to the carbon atom of the methoxy group attached to anthracene, and chemical shift of 65.4 ppm is assigned to the quaternary carbon atom in TAM segment. The asterisks denote the sideband.

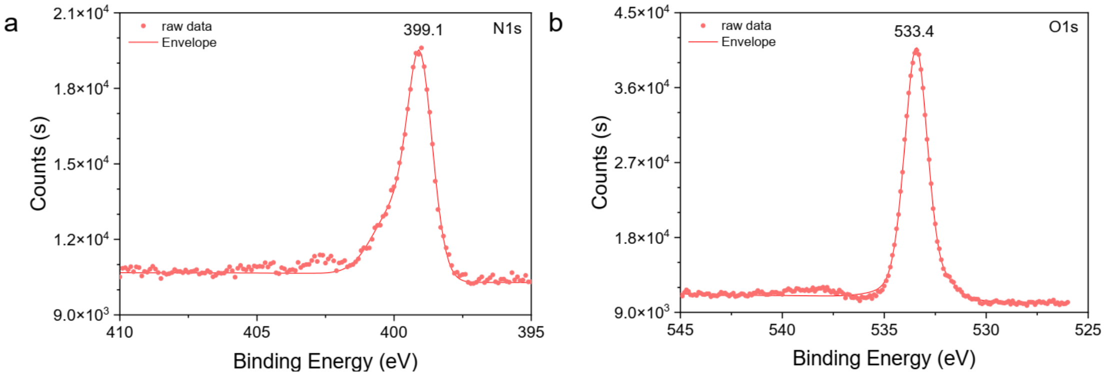

Supplementary Figure 7. High resolution XPS of N 1*s* core level (a), O 1*s* core level (b). The N 1*s* signal peak at 399.1 eV assigned to the sp^2 nitrogen-carbon bond, and the O 1s signal peak at 533.4 eV assigned to sp^3 oxygen-carbon bond.

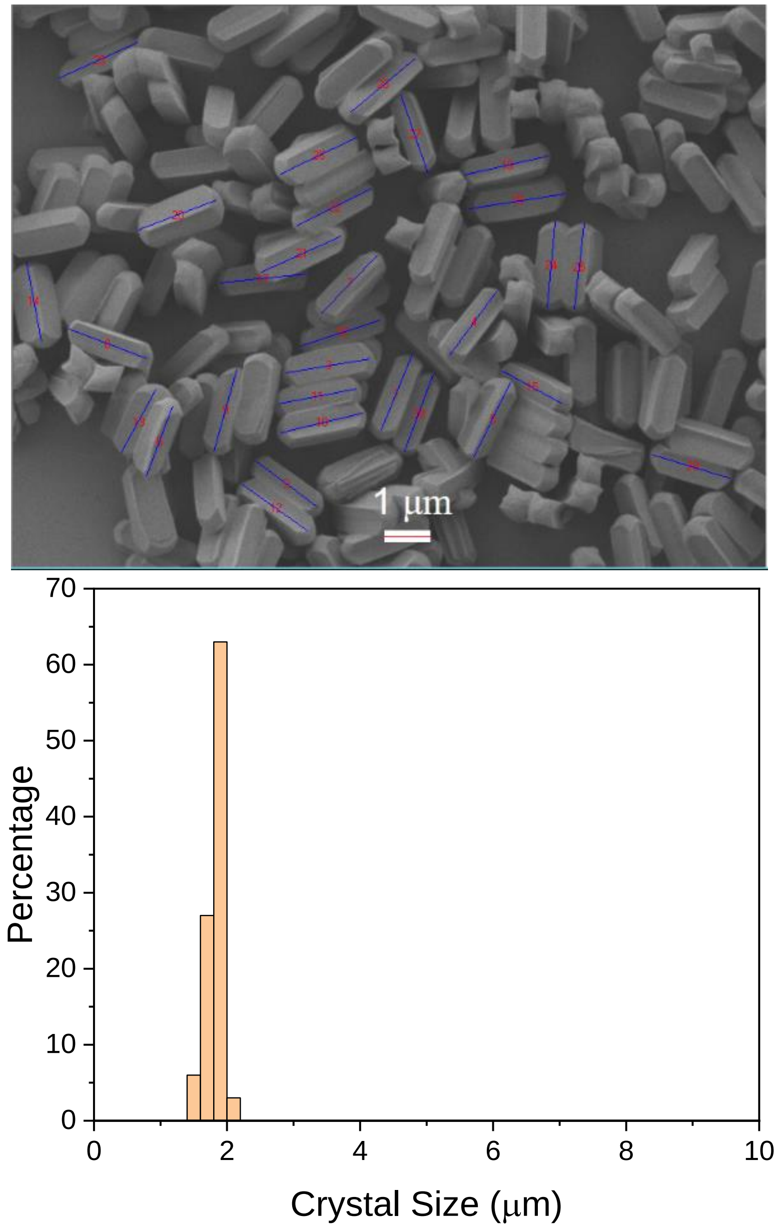

Supplementary Figure 8. SEM image and crystal size distribution of dynaCOF-330. The size of tetragonal prismatic crystals is distributed from 1.5 to 2.1 nm, and over 90% of crystal size is in the range of 1.7-1.9 μm. Statistical crystal size distributions illustrate the uniform morphology of the dyanCOF-330.

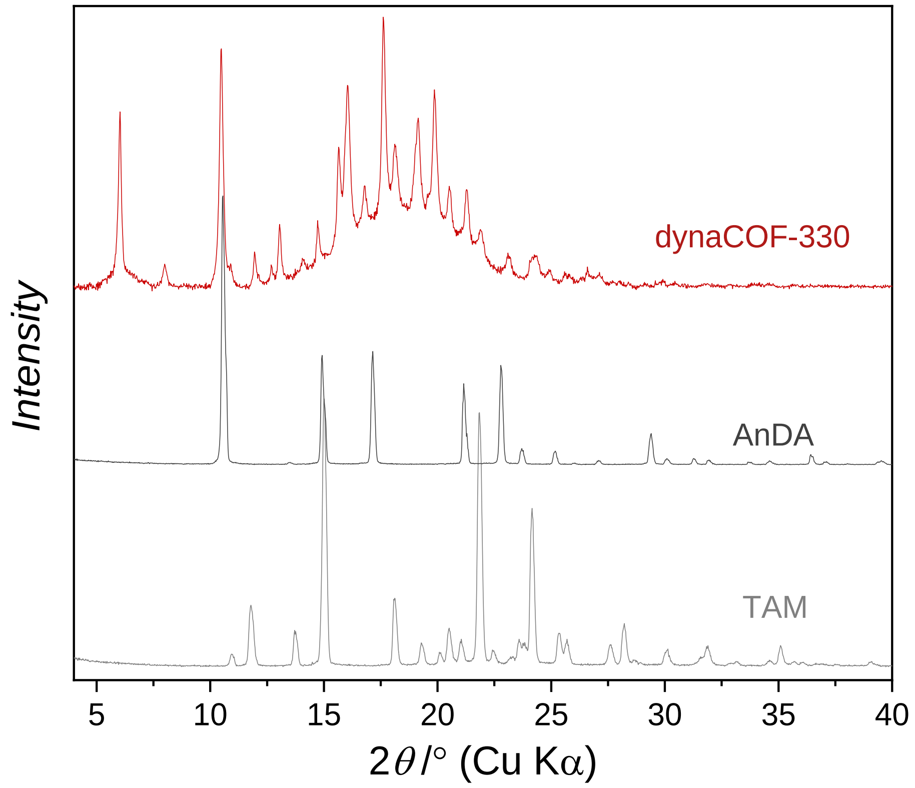

Supplementary Figure 9. PXRD patterns of dynaCOF-330 (red), AnDA (dark grey), and TAM (grey), which illustrate that no AnDA and TAM crystals in the sample of dynaCOF-330.

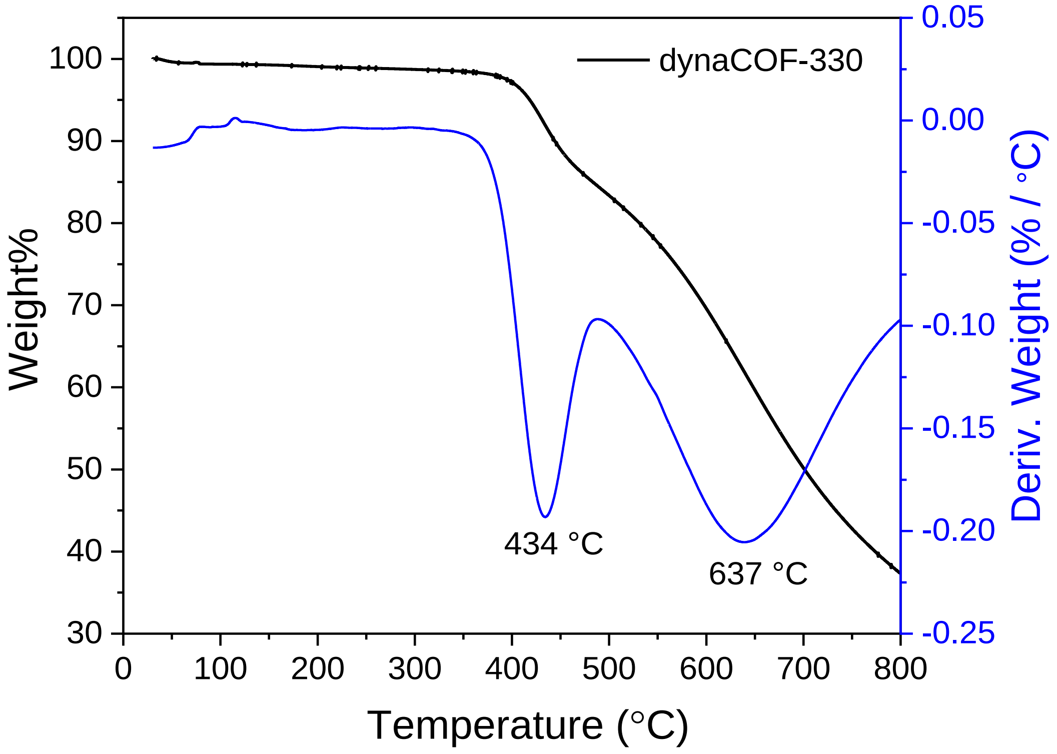

Supplementary Figure 10. The TGA traces of dynaCOF-330 with high thermal stability up to 434 °C.

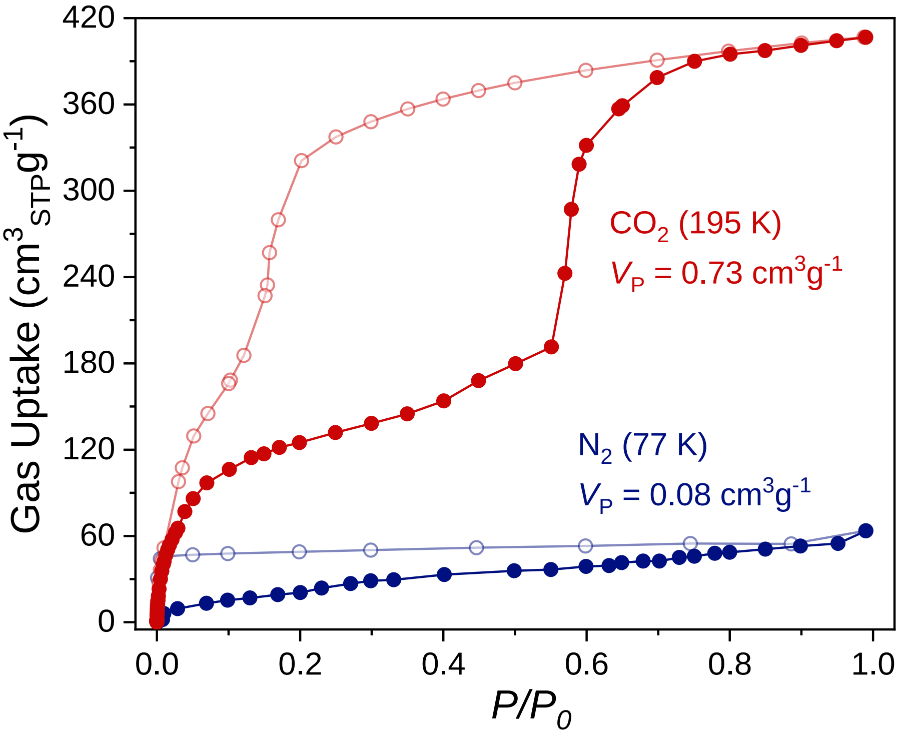

Supplementary Figure 11. The CO_2 and N_2 uptake of dynaCOF-330 at 195 K and 77 K, respectively. Lower N_2 uptake with the pore volume of 0.08 cm^3 g^{-1} is attributed to the lower measurement temperature and weak host-guest interaction. Two distinct steps and a large degree of hysteresis are observed in the CO_2 adsorption isotherm, indicating the possibility of structural dynamics.

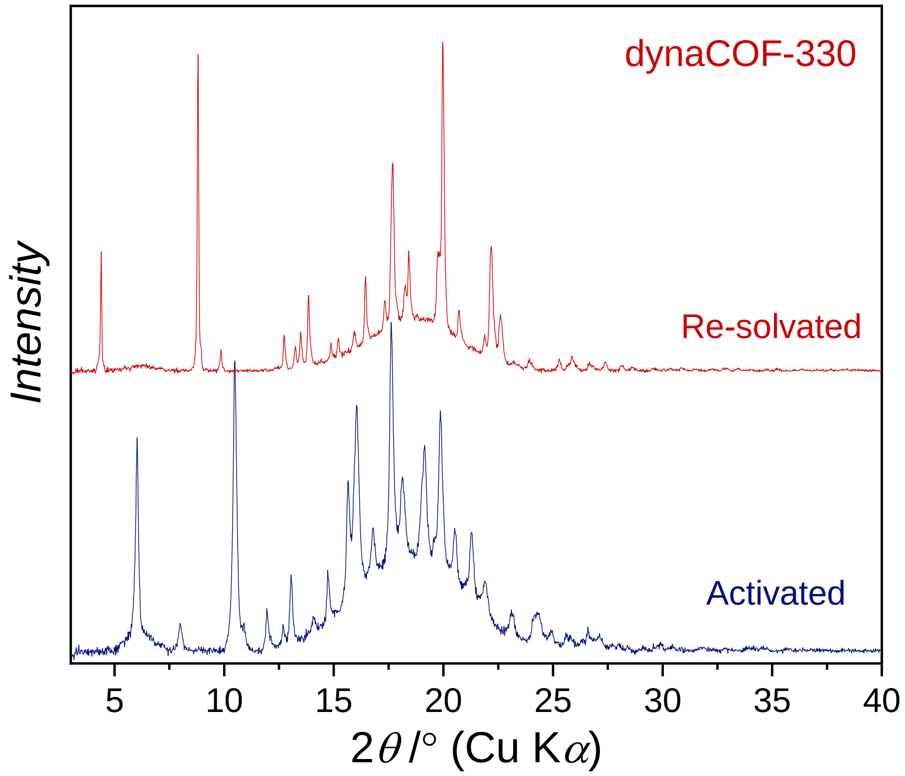

Supplementary Figure 12. PXRD patterns of activated (blue) and re-solvated dynaCOF-330 phase (red). The peak position and intensity both obviously changed for dynaCOF-330 before and after 1,4-dioxane solvation, illustrating the structural dynamics of dynaCOF-330 induced by 1,4-dioxane.

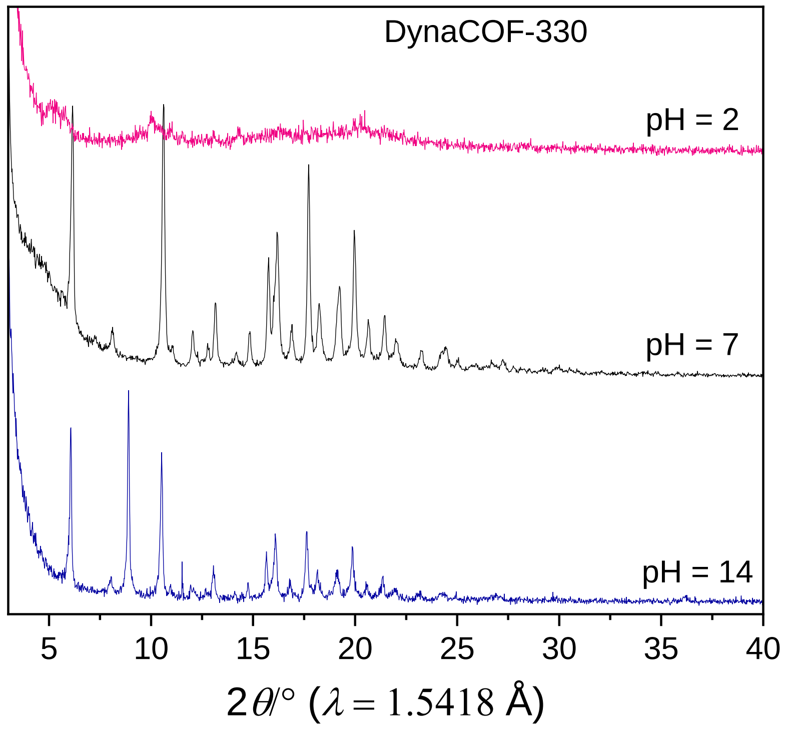

Supplementary Figure 13. PXRD patterns of dynaCOF-330 after treatment in aqueous NaOH solution (pH = 14, blue) and HOAc solution (pH = 2, red) for two hours. The COF is stable in alkali solution but lost long-range ordered structure in acid aqueous.

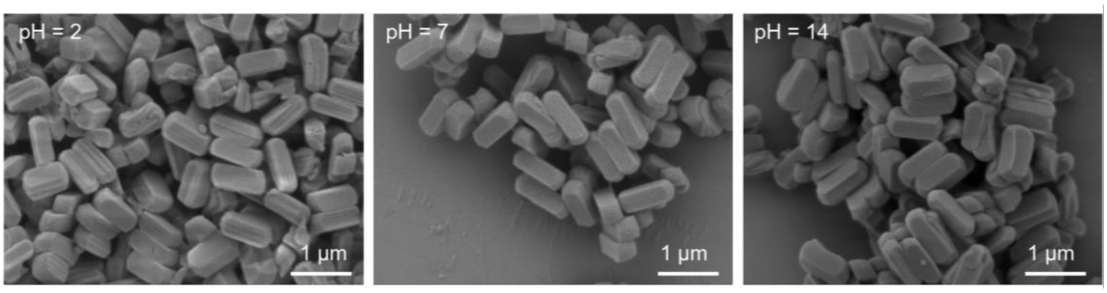

Supplementary Figure 14. SEM images of COF-330 after treatment in various pH aqueous solutions.

Supplementary Section 2. 3D electron diffraction & structural modeling

One drop of 1,4-dioxane dispersed dynaCOF-330 crystals was dropped on a carbon film-supported TEM grid. The grid was put into a high-tilt cryogenic sample holder and then evacuated in a pump station to fully remove the guest molecules. The 3D ED data set was collected on a JEOL JEM2100plus transmission electron microscope (TEM) with the control of the EDT-Collect program. In total, 713 ED frames were recorded with the tilting angle from -59.5° to + 4.9° with the tilting step of 0.10°. Each ED frame was recorded under the spot size 4 with an exposure time of 0.5s. After data processing conducted by the software EDT-Process, 561 reflections were obtained with a resolution close to 2Å.

The 3D reciprocal lattice of dynaCOF-330 was reconstructed and identified as a primitive orthorhombic Bravais lattice with unit cell parameters of a = 17.5 Å, b = 29.8 Å, c = 7.8 Å, and V = 4067.7 Å^3 (Supplementary Figure 15), which were further refined to be a =16.84 Å; b = 29.18 Å; c = 7.64 Å by Pawley refinement against the experimental PXRD pattern. The observed reflection conditions of the 3D ED dataset of dynaCOF-330 were summarized as 0*kl*: $k + l = 2n$; *h*0*l*: $h + l = 2n$; 0*k*0: $k = 2n$; 00*l*: $l = 2n$ (Supplementary Figure 16), which suggests only two possible space groups: *Pnn*2 (no.34) and *Pnnm* (no.58). The potential map generated by Jana2006 software against the 3D ED dataset[2] of dynaCOF-330 also confirms the positions of central carbon atoms of tetrahedral building blocks though the resolution is limited to 2 Å. However, the space group *Pnnm* requires a mirror plane perpendicular to *c* axis (Supplementary Figure 17), which is not geometrically reasonable for both tetrahedral and linear building units. The final determined space group *Pnn*2 was used to build a structural model using Materials Studio software package. To satisfy the *Pnn*2 symmetry, the central carbon atoms of the tetrahedral building blocks were placed at (0, 0, *z*) or (1/2, 0, *z*) corresponding to the position of 2-fold rotation axis. The result of the two choices was almost the same except for a 1/2 shift of unit cell origin along *a* axis.

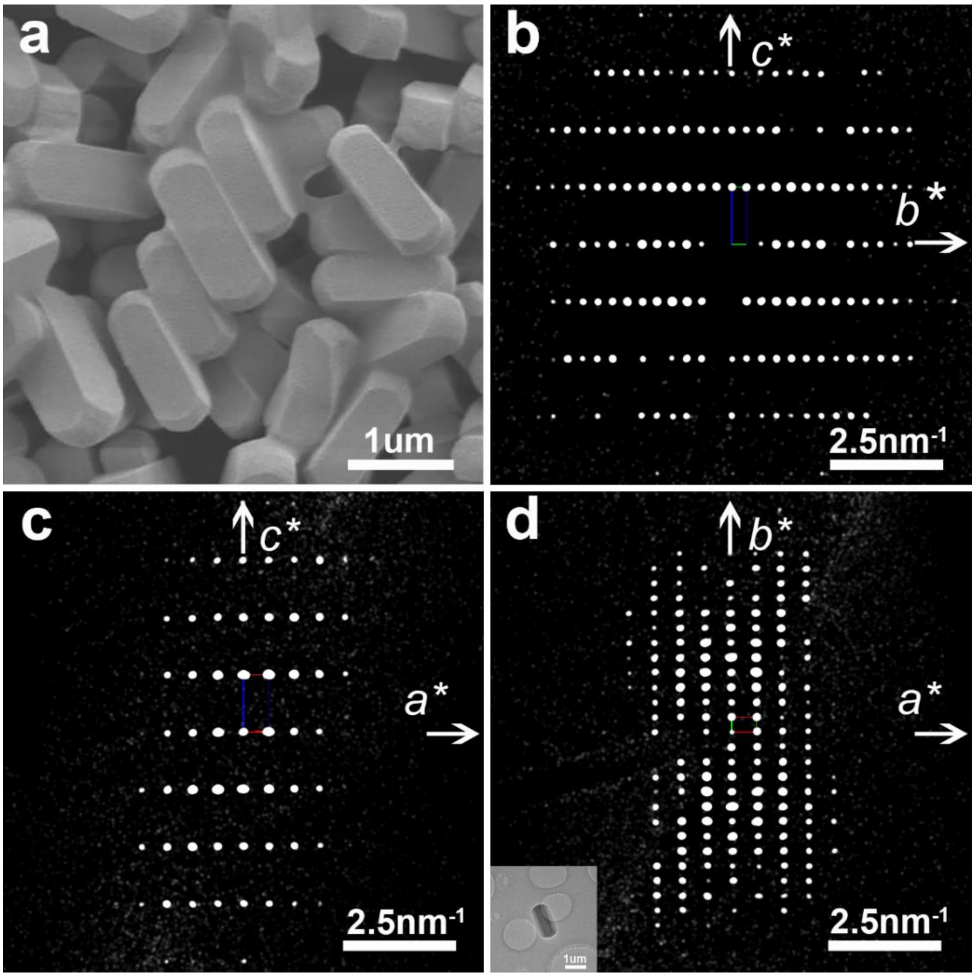

Supplementary Figure 15. (a)SEM image of high crystalline dynaCOF-330 crystals. Projections of reconstructed three-dimensional reciprocal lattice of dynaCOF-330 along (b) [100], (c) [010] and (d) [001] directions. The intensity of every pixel has been extracted the square root to show the week diffraction points more clearly. The unit cell parameter of a = 17.5 Å, b = 29.8 Å, c = 7.8 Å, $\alpha = \beta = \gamma = 90^{\circ}$ and V = 4067.7 Å^3 was identified.

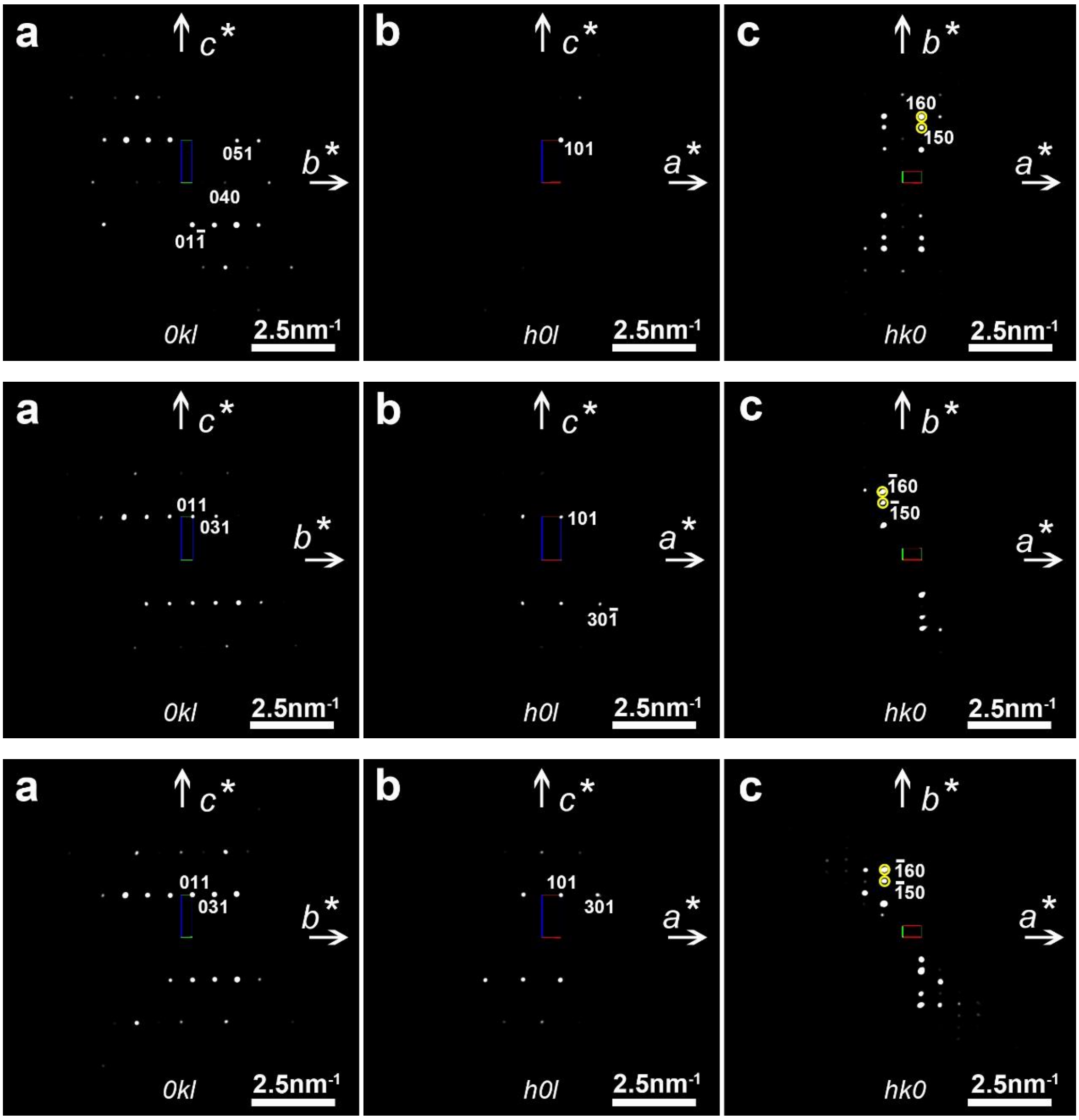

Supplementary Figure 16. (a) (0*kl*), (b) (*h*0*l*) and (c) (*hk*0) slices cut from reconstructed three-dimensional reciprocal lattice of dynaCOF-330 (three 3D electron diffraction data set). The reflection conditions were summarized as: 0*kl*: $k + l = 2n$; *h*0*l*: $h + l = 2n$; 0*k*0: $k = 2n$; 00*l*: $l = 2n$, suggesting two possible space groups – *Pnnm* (No.58), *Pnn*2 (No.34).

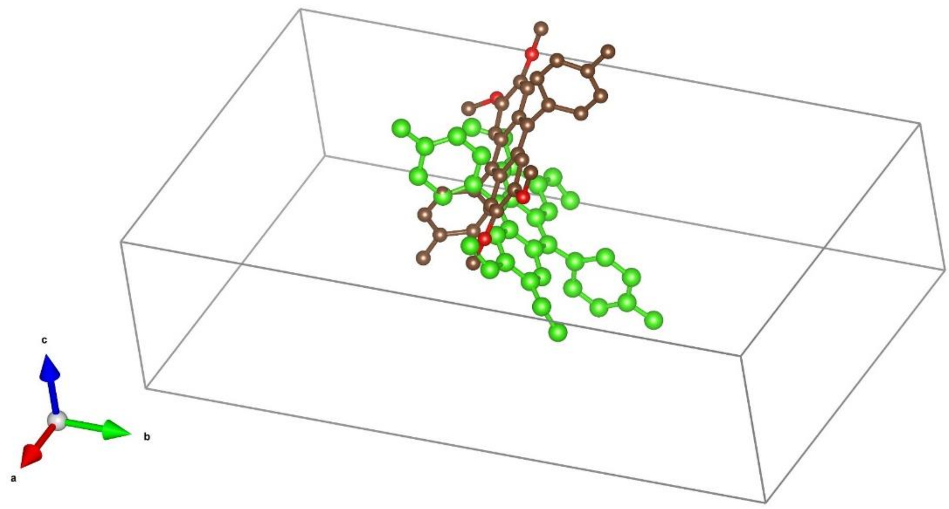

Supplementary Figure 17. The *Pnnm* space group requires a mirror plane perpendicular to *c* axis which will generate symmetry-related fragments (marked as green color). The brown and red color atoms in the original fragment are C and O elements, respectively. The fragment generated by the mirror plane intersects with the original one. It indicates the structure model is not geometrically robust.

3D electron diffraction data of guest contained sample. For the structure determination of guest adsorbed samples, we first examined the sample dispersed in 1,4-dioxane solvent followed by the CryoEM fast freezing protocol[3]. That means 1,4-dioxane solvent outside dynaCOF-330 crystals may be solidified into amorphous solid while 1,4-dioxane molecules in the channel of dynaCOF-330 crystals are well stabilized. However, strong diffractions of 1,4-dioxane crystals were observed during the 3D electron diffraction data collection which hinders the reflections extraction of dynaCOF-330. We finally tried to prepare the guest adsorbed sample by fumigation (Supplementary Figure 18).

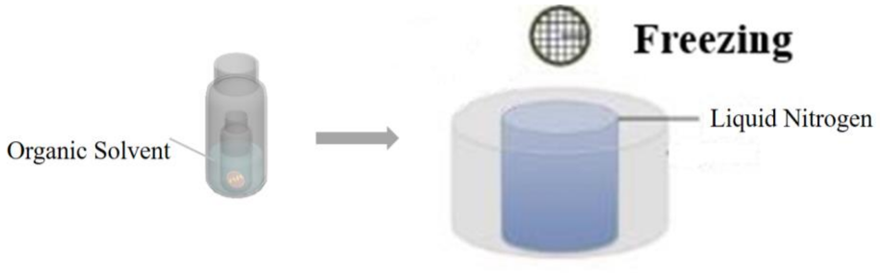

Supplementary Figure 18. The fumigation process of guests contained sample preparation for 3D electron diffraction data collection.

The 3D electron diffraction data of dynaCOF-330-1,4-Dioxane was collected with a condition similar to dynaCOF-330-Activated. The reconstructed reciprocal lattice of dynaCOF-330 shows a primitive tetragonal Bravais lattice with the unit cell parameter of $a = b = 27.2$ Å, $c = 7.9$ Å, $\alpha = \beta = \gamma = 90^\circ$ and $V = 5844.7$ Å^3 (Supplementary Figure 19). The observed reflection conditions from 3D electron diffraction data were summarized as: $hk0$: $h + k = 2n$, $00l$: $l = 2n$. PXRD analysis was then performed based on the unit cell and reflection conditions of 3D electron diffraction data of dynaCOF-330-expanded, which suggest a unit cell parameter of $a = b = 27.84$ Å, $c = 7.26$ Å, $\alpha = \beta = \gamma = 90^\circ$. An extra reflection, $0k0$: $k = 2n$, was observed in the PXRD pattern, which was not recorded in the 3D electron diffraction data due to the limited sampling area of reciprocal space. The observed reflection conditions suggest only one possible space group: $P4_2/n$ (No.86). A 10-fold interpenetrated structural model was then successfully built based on the refined unit cell parameter and space group of $P4_2/n$.

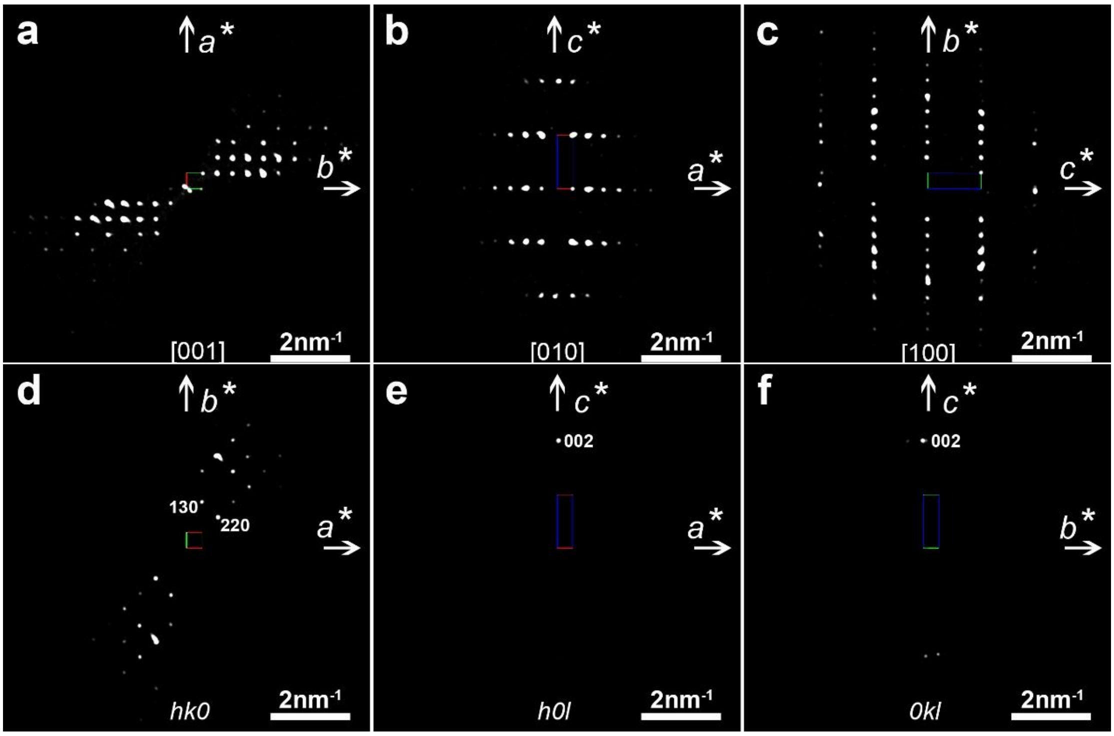

Supplementary Figure 19. (a), (b) and (c) Projections of reconstructed reciprocal lattice along [001], [010] and [100] directions respectively. The reciprocal lattice suggested a tetragonal primitive Bravais lattice with unit cell parameters of $a = b = 27.2$ Å, $c = 7.9$ Å, $V = 5844.7$ Å^3. (d), (e) and (f) *hk*0, *h*0*l*, 0*kl* slices of the reciprocal lattice. The observed reflection conditions can be summarized as *hk*0: $h + k = 2n$, 00*l*: $l = 2n$, which are consistent with the PXRD pattern.

The central carbon atom positions of the tetrahedral building block can be located from this potential map. Compared to the constructed structure model, the origin of the unit cell in this potential map shifts 1/2 alone the *a* axis.

To obtain a reasonable framework, the possible structure naturally follows the **dia**-c*N* topology. Three possible candidate structure models with the interpenetration of 6-fold, 10-fold, and 14-fold were exanimated (Supplementary Figure 20) [4]. The result shows that the length between two adjacent central carbon atoms of the 6-fold structural model is too short while the 14-fold one is very long. At the same time, the 14-fold interpenetrated structure model is too crowded which indicates that the structure is not geometrically robust. Therefore, the 10-fold interpenetrated structural model was determined to be a proper choice. During the construction of 10-fold interpenetrated structure model, we found that the adjacent linear linking units are still a little bit close to each other if no fragment distortion was involved. This indicates that the structure needs to be distorted a little bit to ensure a more reasonable geometry. Then the generated model was further refined by Rietveld refinement against PXRD intensity. The final structure of dynaCOF-330 is identified to adopt a 10-fold interpenetrated **dia** topology with *Pnn*2 space group.

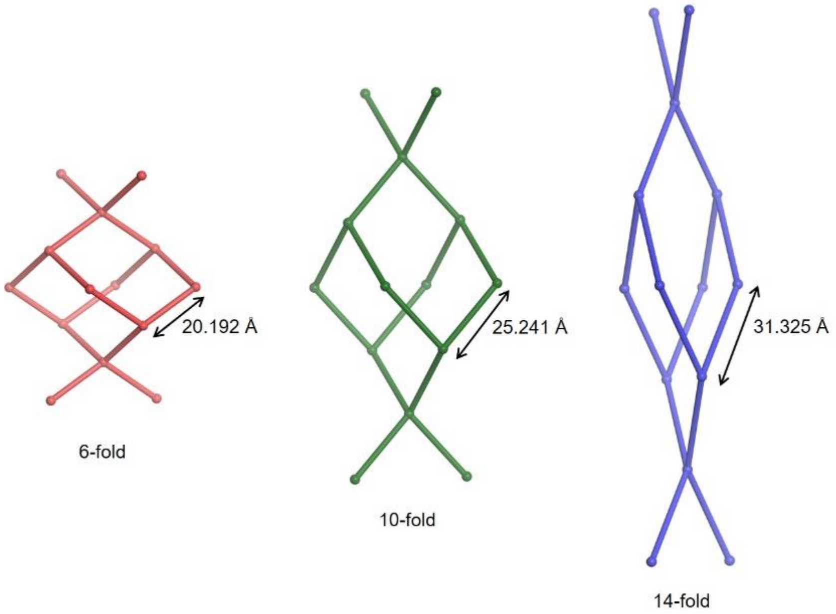

Supplementary Figure 20. Cages of **dia** topology with interpenetration of 6-fold, 10-fold, and 14-fold along *c* axis. The three possible candidates were built and exanimated based on refined unit cell parameters of dynaCOF-330.

Supplementary Section S3. Synchrotron powder X-ray diffraction analyses

Powder X-ray diffraction (PXRD) data were collected on a Bruker D8 Advance diffractometer with Cu Kα radiation. The high-resolution PXRD patterns were collected at the beamline BL14B1 of the Shanghai Synchrotron Radiation Facility (SSRF) using X-ray with a wavelength of 0.6895 Å and transition model [5]. The samples were held in a glass capillary and kept spinning during the measurement.

DynaCOF-330 was filled in the borosilicate glass capillary with an outer diameter of 0.8 mm and thickness of 0.01 mm, which is continuously spinning during the experiments to improve data statistics. The monochromatic X-ray beam with an energy of 18 keV (λ = 0.6895 Å) and a beam size of 180 μm (width) × 200 μm (height) was adopted. The Mythen 1K linear detector was adopted for high-resolution powder diffraction data acquisition in Debye-Sherrer mode. The wavelength of the X-ray was calibrated using the LaB_6 standard from NIST(660b) [6]. The transmission in PXRD patterns was collected at room temperature. Sample in the capillary is about 2 cm in length. Before vapour dosing, overnight degassing was needed. At each selected pressure, it took 4 hours at least for the system to reach equilibrium. No thermostat is used during vapour dosing, but the temperature was monitored with a thermometer to ensure the selected relative pressure was unchanged. Vapour dosing and pressure controlling were performed by BELSORP-max.

PXRD refinement. The indexing of the PXRD patterns, as well as Pawley refinement, was performed using the Reflex module in Materials Studio 2017[7]. The initial structure of dynaCOF-330 and dynaCOF-330IV (dioxane) were determined by using 3D ED and model building strategy (Supplementary Section 2). PXRD patterns of dynaCOF-330III (dioxane) and dynaCOF-330IV (acetone) show the same diffraction law with dynaCOF-330IV (dioxane), suggesting they are adopting the same space group of $P4_2/n$. PXRD patterns of dynaCOF-330I (dioxane) and dynaCOF-330I (acetone) show almost identical diffraction peak positions but different peak intensities which suggests that they are adopting the same space group of *Pnn*2 and similar empty framework structures. Unit cell parameters of dynaCOF-330III (acetone) were determined by indexing using the X-Cell program. With enough diffraction peaks (over

fifty) of dynaCOF-330III (acetone) and the characteristic unit cell parameter for this kind of COFs with one axis of about 8 Å, the unique reasonable space group of *P*2/*n* and unit cell parameters of a = 22.17 Å, b = 7.32 Å, c = 32.50 Å, $\alpha = \gamma = 90°$, $\beta = 92.92°$ were determined.

DynaCOF-330 adopts a 10-fold interwoven structure, and we think it is impossible to change the number of interweaving for the evolution of crysal structure during the rapid (~ 1 s) guest inclusion process. Therefore, the interwoven number is consistent for all the structural models. Before refinement, a structural model was built in Material Studio software with a molecular fragment extracted from the molecular analog crystal of dynaCOF-330 (Supplementary Figure 4). The background of PXRD pattern was firstly removed to make the refinement process more straightforward. The structure model was constrained by bond lengths and angles. The atomic displacement parameters (ADPs) of the same element were constrained to be equal and the Biso value was limited to between 1 and 10. After the refinement of the empty framework, guest molecules were added to the unit cell and refined using the same procedure, but keeping the framework atoms rigid. The number of added guest molecules was calculated from the adsorption isotherm. Molecular conformations of dynaCOF-330 before and after guest uptake are shown in Supplementary Figure 21 and Supplementary Figure 26.

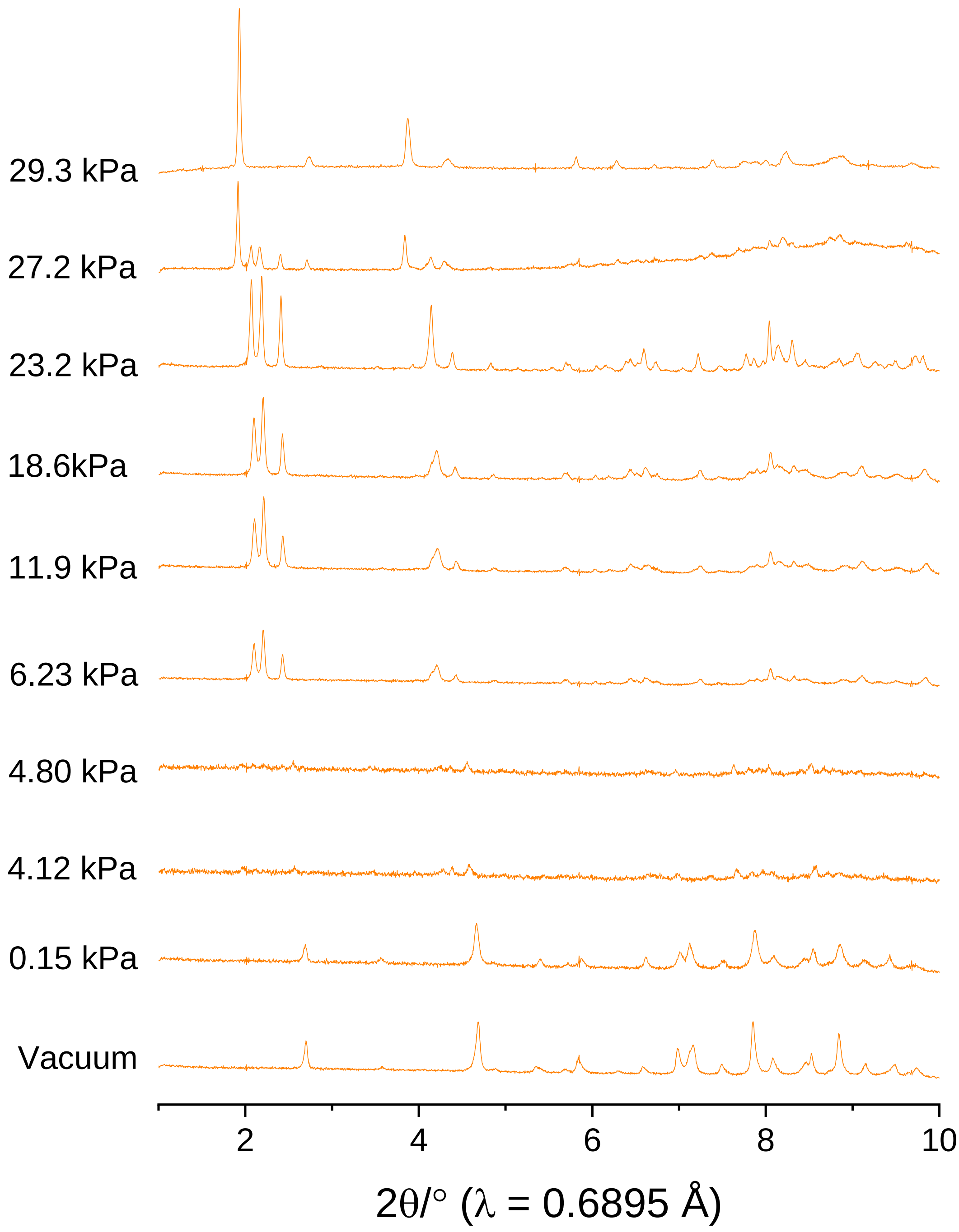

Supplementary Figure 21. PXRD patterns of dynaCOF-330 were obtained at the corresponding acetone vapor pressures. There are three crystalline stages and the PXRD pattern of dynaCOF-330 at acetone vapour of 27.2 kPa is a mixed phase of vapour pressure at 23.2 and 29.3 kPa. Indexing of the collected PXRD patterns indicates that there are three crystalline phases and their lattice constants are shown in Supplementary Table 1.

Supplementary Table 1. Crystallographic data on *ex-situ* PXRD was measured during the adsorption of acetone at room temperature.

**Acetone vapour Pressure (kPa)**	***a***	***b***	***c***	α	β	γ
Vac	16.9	29.22	7.67	90	90	90
0.15	16.96	29.22	7.56	90	90	90
4.80	22.11	7.32	32.50	90	93.12	90
6.23	22.12	7.33	32.55	90	93.09	90
11.9	22.17	7.32	32.50	90	92.92	90
18.6	22.34	8.00	32.52	90	93.13	90
23.2	22.31	7.95	32.46	90	93.08	90
27.2	29.13	29.13	7.17	90	90	90
29.3	28.78	28.78	7.2	90	90	90

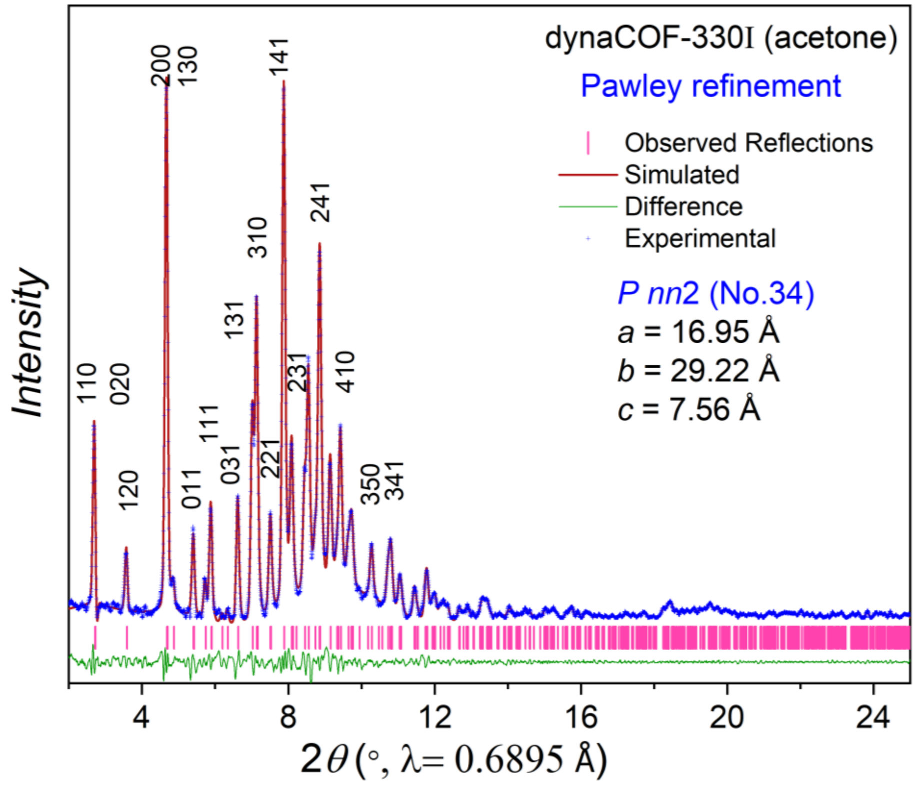

Supplementary Figure 22. Pawley refinement of the synchrotron PXRD data of dynaCOF-330I (acetone). This PXRD pattern was collected by exposing the COF sample to acetone vapor pressure of 0.15 kPa. The diffraction law of dynaCOF-330I (acetone) is the same as dynaCOF-330. Therefore, dynaCOF-330 is adopted as the initial empty structure for PXRD refinement of dynaCOF-330I (acetone), and then two guest molecules were introduced per unit cell which is determined according to the acetone vapor adsorption isotherm. The final R_{wp} and R_p values converged to be 5.27% and 7.89%.

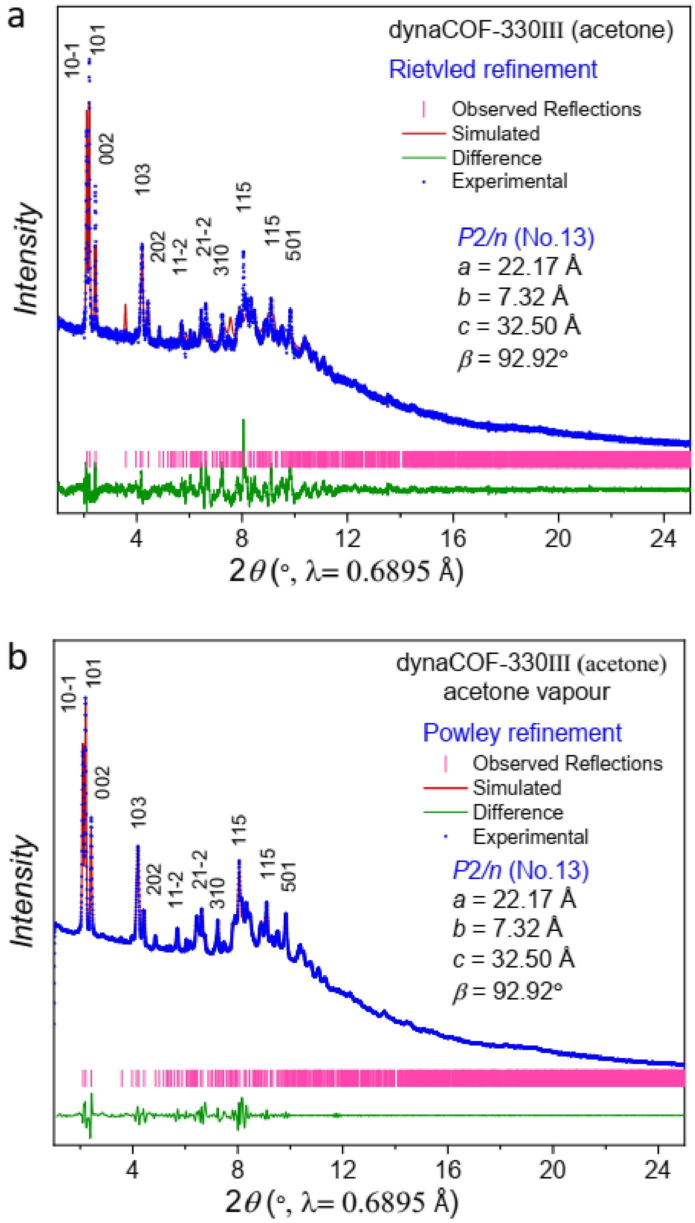

Supplementary Figure 23. Pawley refinement (a) and Rietveld refinement (b) of the synchrotron PXRD data of dynaCOF-330III (acetone). The PXRD pattern was collected by exposing the COF sample to acetone vapor of 11.6 kPa. According to the acetone vapor adsorption isotherm, sixteen acetone molecules are introduced in each unit cell to construct starting structural model. After Rietveld refinement, the simulated peak intensity at low diffraction angle is matched well with the experiment data. However, at high diffraction angle, the difference is obvious which is probably caused by the movement of guests in the channel. The Pawley refinement showed the simulated peaks matched well with the experimental one.

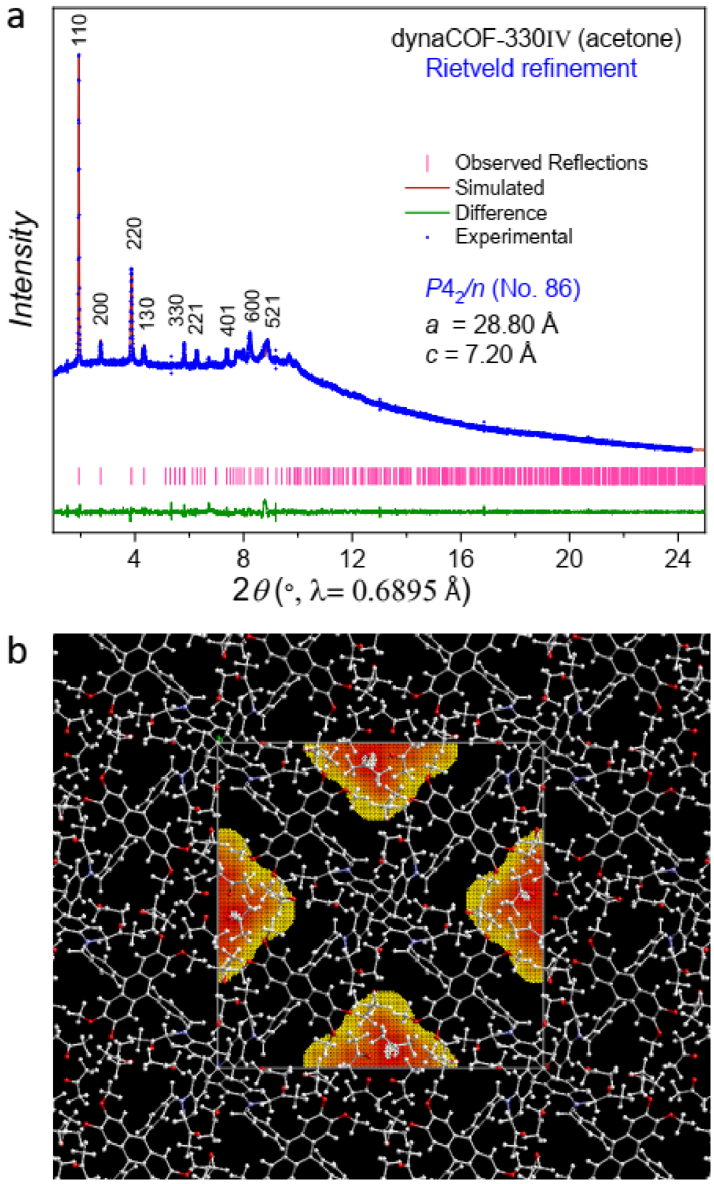

Supplementary Figure 24. Rietveld refinement of the synchrotron PXRD data (a) and Fourier difference diagram (b) of dynaCOF-330IV (acetone). The PXRD pattern was collected by exposing the COF sample to saturated acetone vapor of 30 kPa. Nineteen acetone molecules are introduced in each unit cell of dynaCOF-330 to construct the initial structural model for PXRD refinement, the number of guests is determined according to the acetone vapor adsorption. The final R_{wp} and R_p values converged to be 1.98% and 1.46%. Fourier difference map (Fo-Fc) by setting the occupancy of guests as zero shows the guests located at the position having residual electron density. However, limited by the low resolution of PXRD data, the orientation of guests cannot be determined.

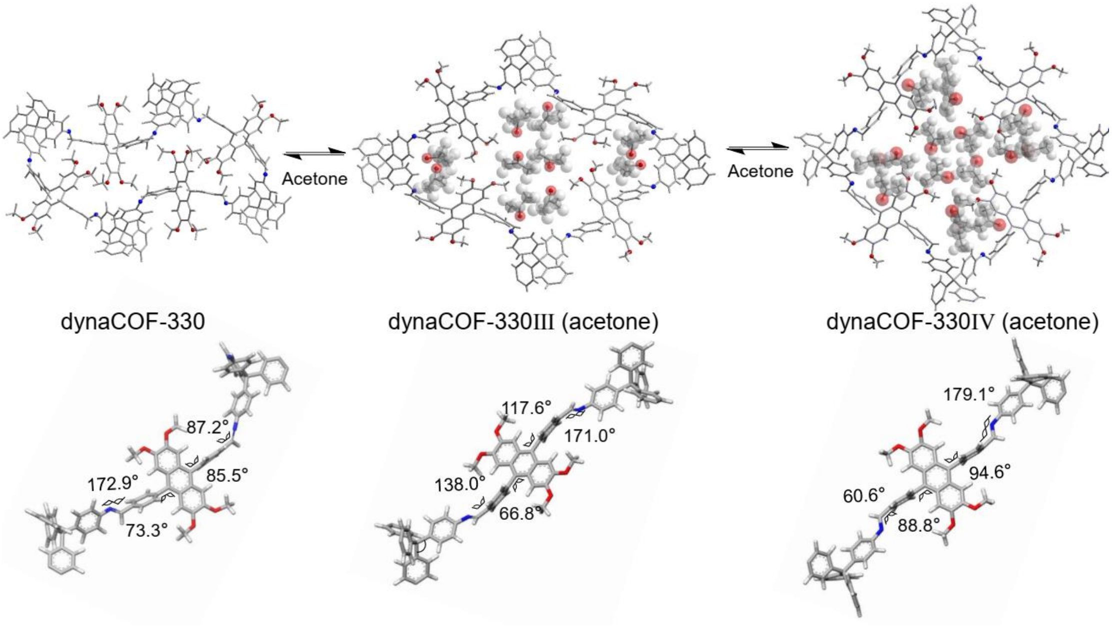

Supplementary Figure 25. Molecular conformations of dynaCOF-330, dynaCOF-330III (acetone), and dynaCOF-330IV (acetone).

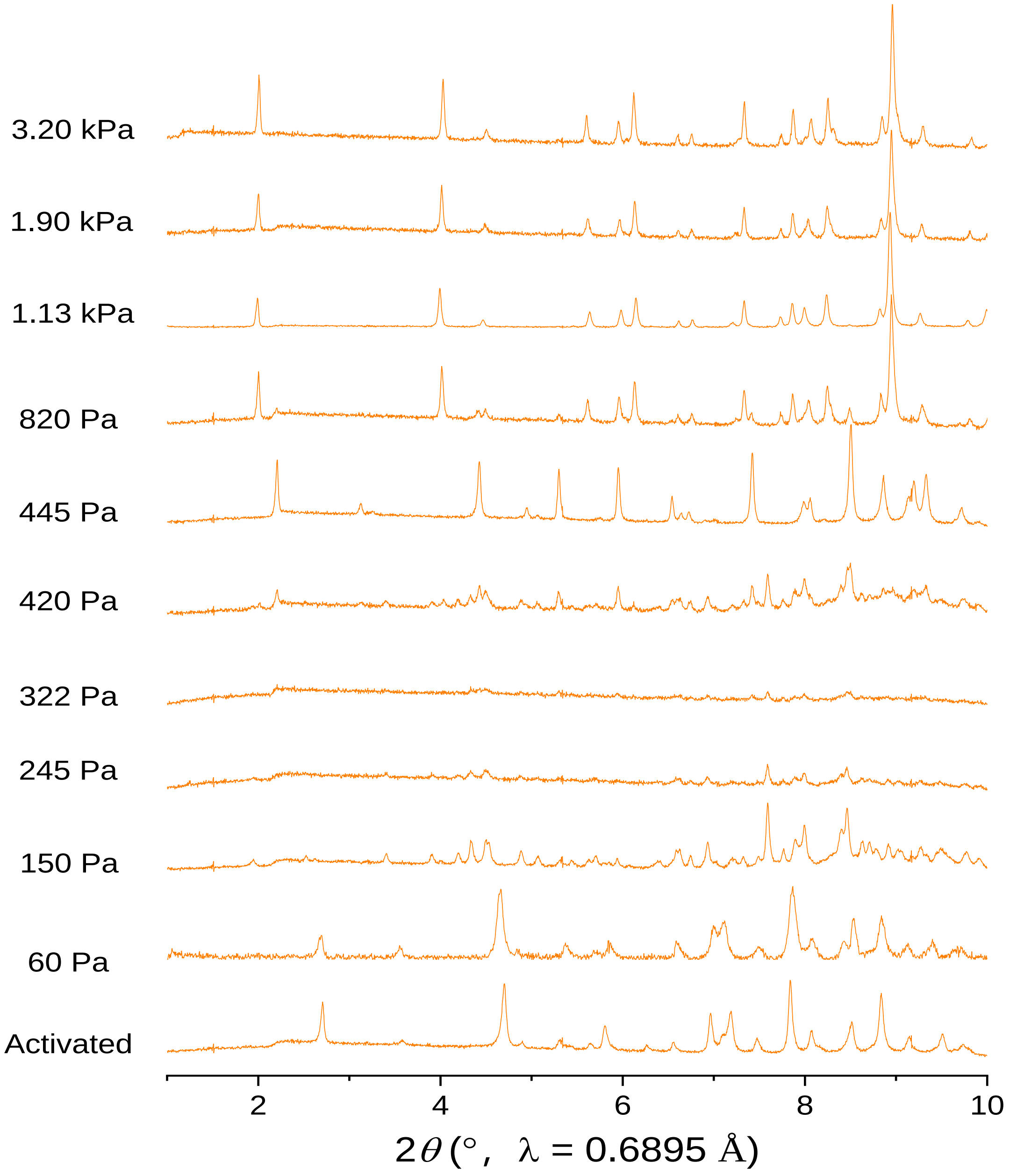

Supplementary Figure 26. PXRD patterns of dynaCOF-330 were obtained at the corresponding 1,4-dioxane vapor pressures. PXRD pattern of dynaCOF-330 at 820 Pa is a mixed phase illustrating the intermediate phase has not fully evolve` to the expanded phase. Indexing of the collected PXRD patterns indicates that there are three crystalline phases and their lattice constants are shown in Supplementary Table 2.

Supplementary Table 2. Crystallographic data on *ex-situ* PXRD was measured during the adsorption of 1,4-dioxane at room temperature.

**1,4-dioxane vapour Pressure (kPa)**	***a***	***b***	***c***	α	β	γ
Vac	16.85	29.21	7.62	90	90	90
0.06	17.03	29.3	7.58	90	90	90
0.42	25.10	25.10	8.00	90	90	90
0.445	24.96	24.96	8.00	90	90	90
0.82	27.81	27.81	7.54	90	90	90
1.13	27.97	27.97	7.49	90	90	90
1.9	27.83	27.83	7.53	90	90	90
3.2	27.73	27.73	7.53	90	90	90

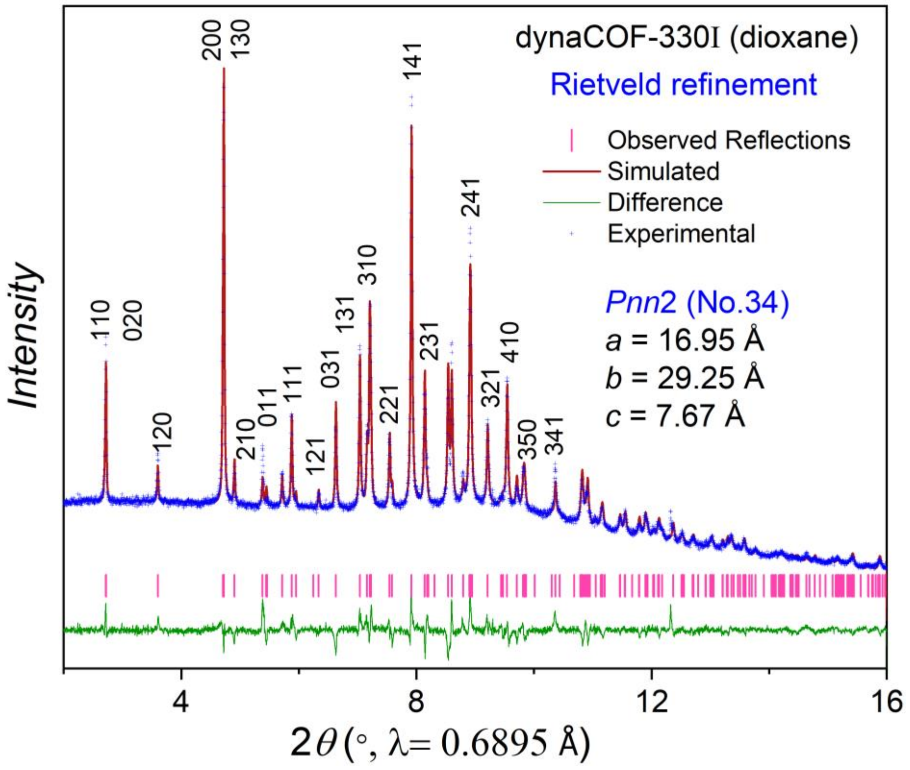

Supplementary Figure 27. Rietveld refinement of the synchrotron PXRD data of dynaCOF-330I (dioxane). This PXRD pattern was collected by exposing the COF sample to 1,4-dioxane vapor pressure of 0.06 kPa. There are two guest molecules introduced per unit cell. The final R_{wp} and R_p values converged to be 4.32% and 1.89%.

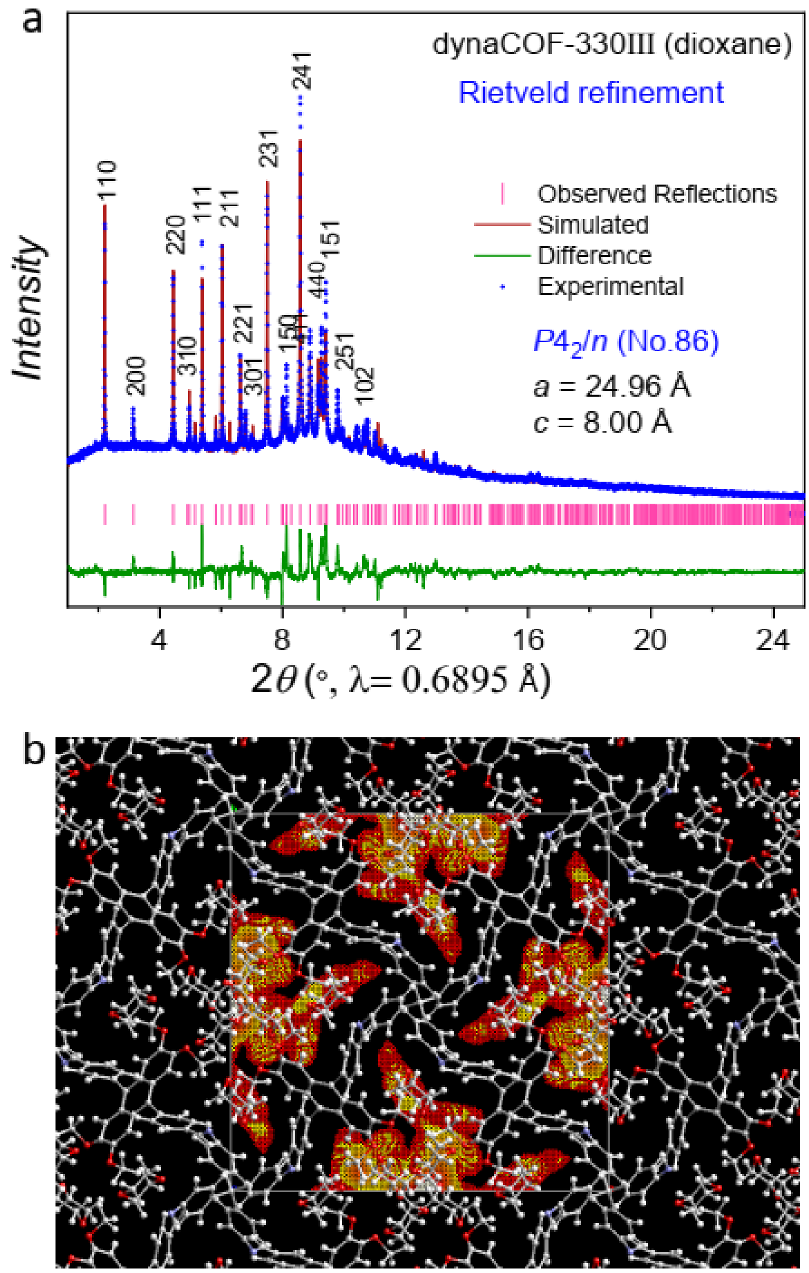

Supplementary Figure 28. Rietveld refinement of the synchrotron PXRD data (a) and Fourier difference diagram (b) of dynaCOF-330III (dioxane). The PXRD pattern was collected at 0.445 kPa of 1,4-dioxane vapor, at which twelve 1,4-dioxane molecules were adsorbed into each unit cell, according to the 1,4-dioxane vapour uptake isotherm. The final R_{wp} and R_p values converged to be 7.58% and 5.25%. Fourier difference map (Fo-Fc) by setting the occupancy of guests as zero shows the guests located at the position having residual electron density indicating the refined structural model is reasonable.

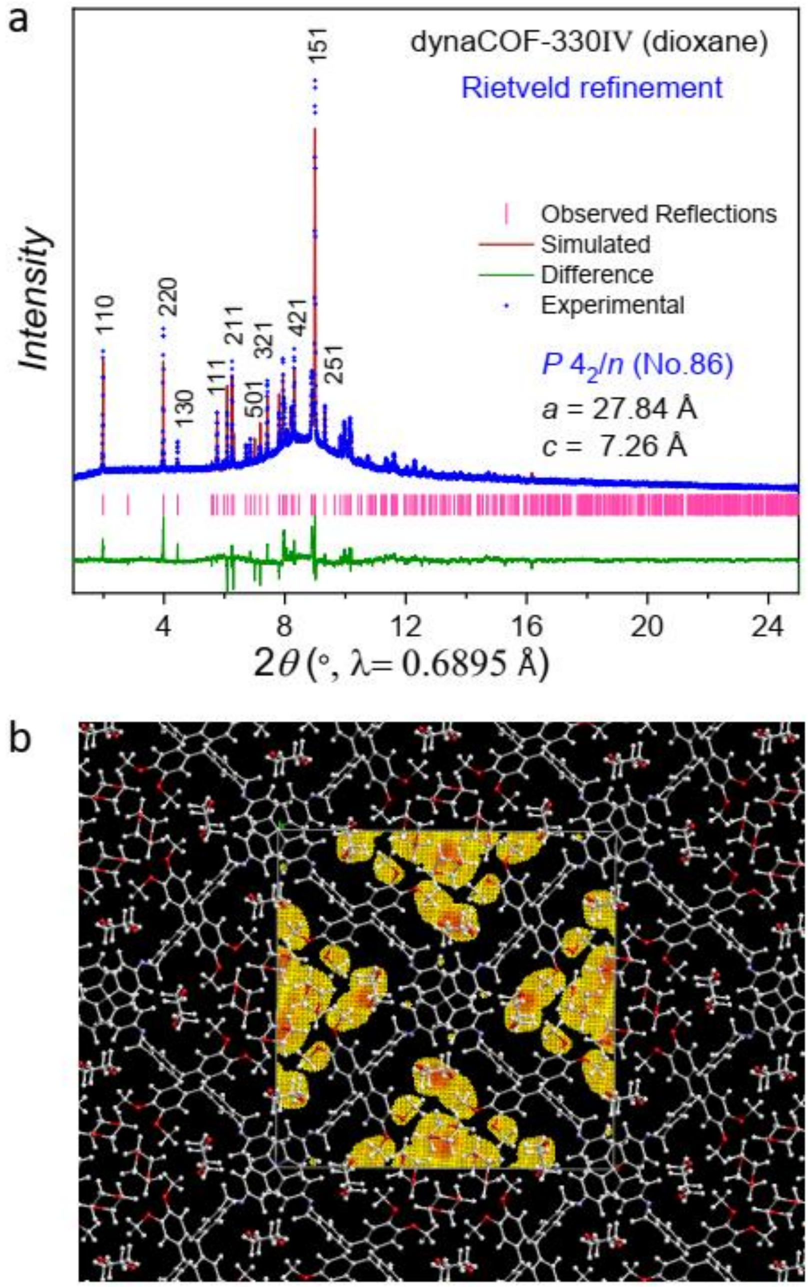

Supplementary Figure 29. Rietveld refinement of the synchrotron PXRD data (a) and Fourier difference diagram (b) of dynaCOF-330IV (dioxane). This PXRD pattern was collected at 1.9 kPa of 1,4-dioxane vapour. The only possible space group of $P4_2/n$ was determined by EDT data and PXRD analysis (Supplementary Figure 19). There are sixteen 1,4-dioxane molecules adsorbed into each unit cell of the initial structural model for PXRD refinement. The guest number is determined by the 1,4-dioxane vapour adsorption isotherm. The final R_{wp} and R_p values converged to be 7.86% and 5.47%. The Fourier difference map (Fo-Fc) shows the location of guests.

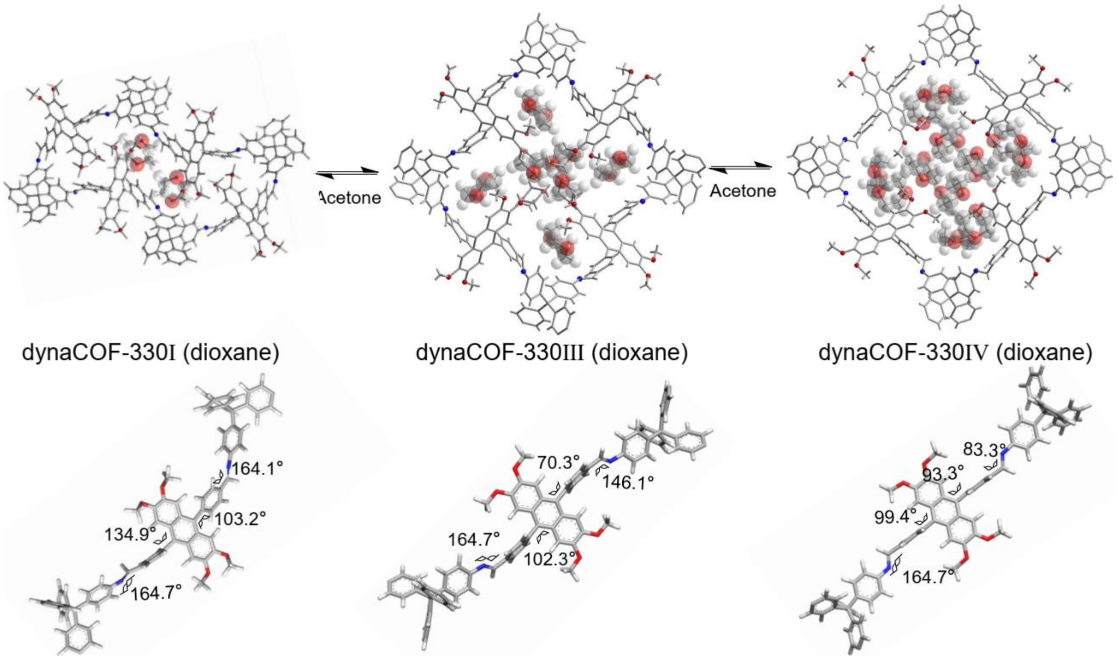

Supplementary Figure 30. Molecular conformations of dynaCOF-330I (dioxane), dynaCOF-330III (dioxane), and dynaCOF-330IV (dioxane).

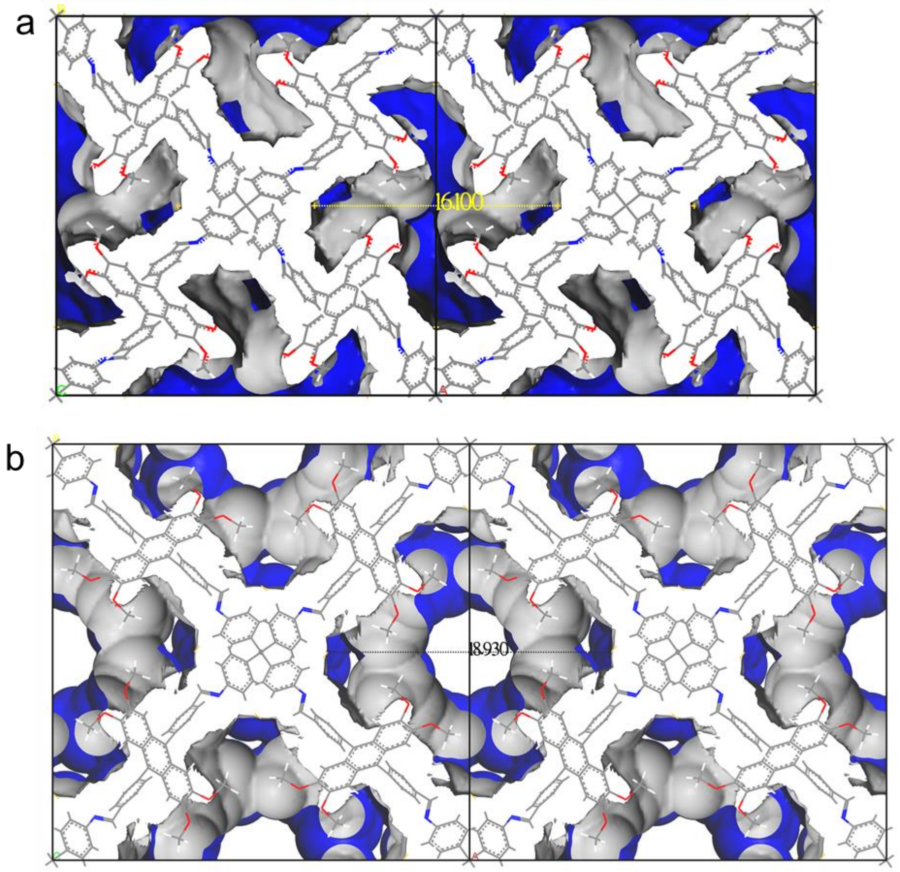

Supplementary Figure 31. Channel sizes and shapes of dynaCOF-330III(dioxane)(a) and dynaCOF-330IV(dioxane)(b).

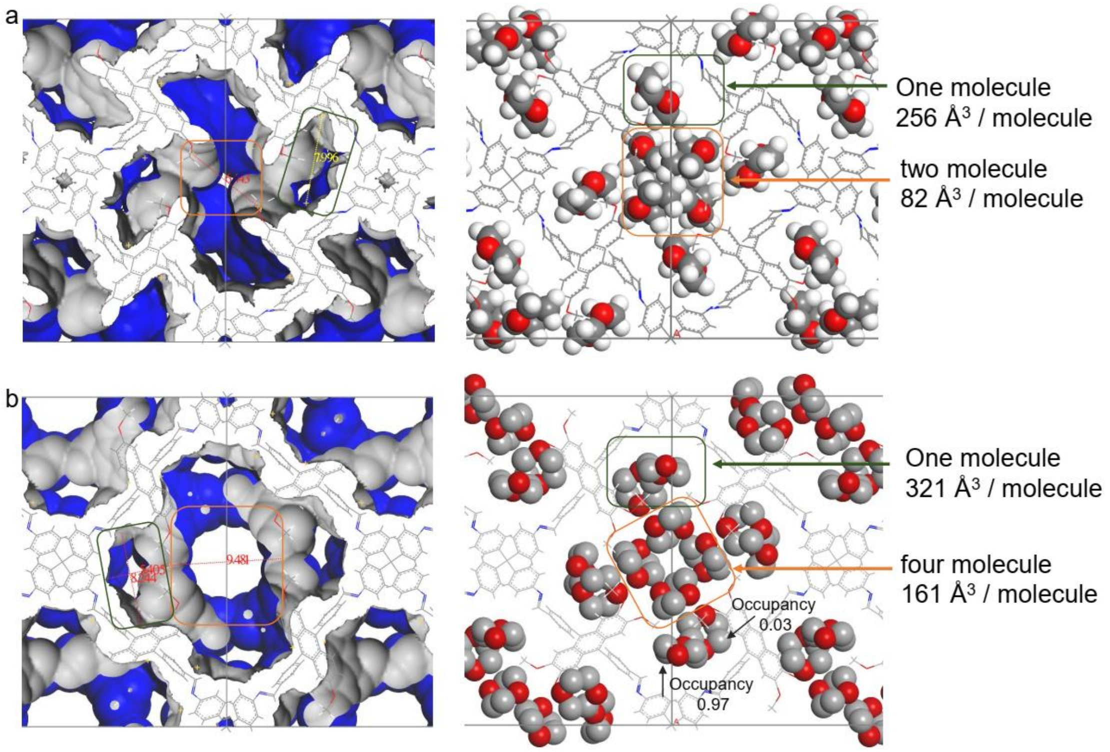

Supplementary Figure 32. Schematic illustration of guest redistribution during 1,4-dioxane uptake of dynaCOF-330III (dioxane) (a) and dynaCOF-330IV (dioxane) (b). In the channel center, one 1,4-dioxane molecule adopts an average volume of 82 Å^3 in the channel center for dynaCOF-330III (dioxane) and 161 Å^3 for dynaCOF-330IV (dioxane), one 1,4-dioxane molecule adopts an average volume of 161 Å^3 in the channel center and 256 Å^3 in the corner, respectively. High spatial occupancy for dynaCOF-330III (dioxane) the guests located in the center can more efficiently hinder the vibration of fluorophores and display higher fluorescence intensity.

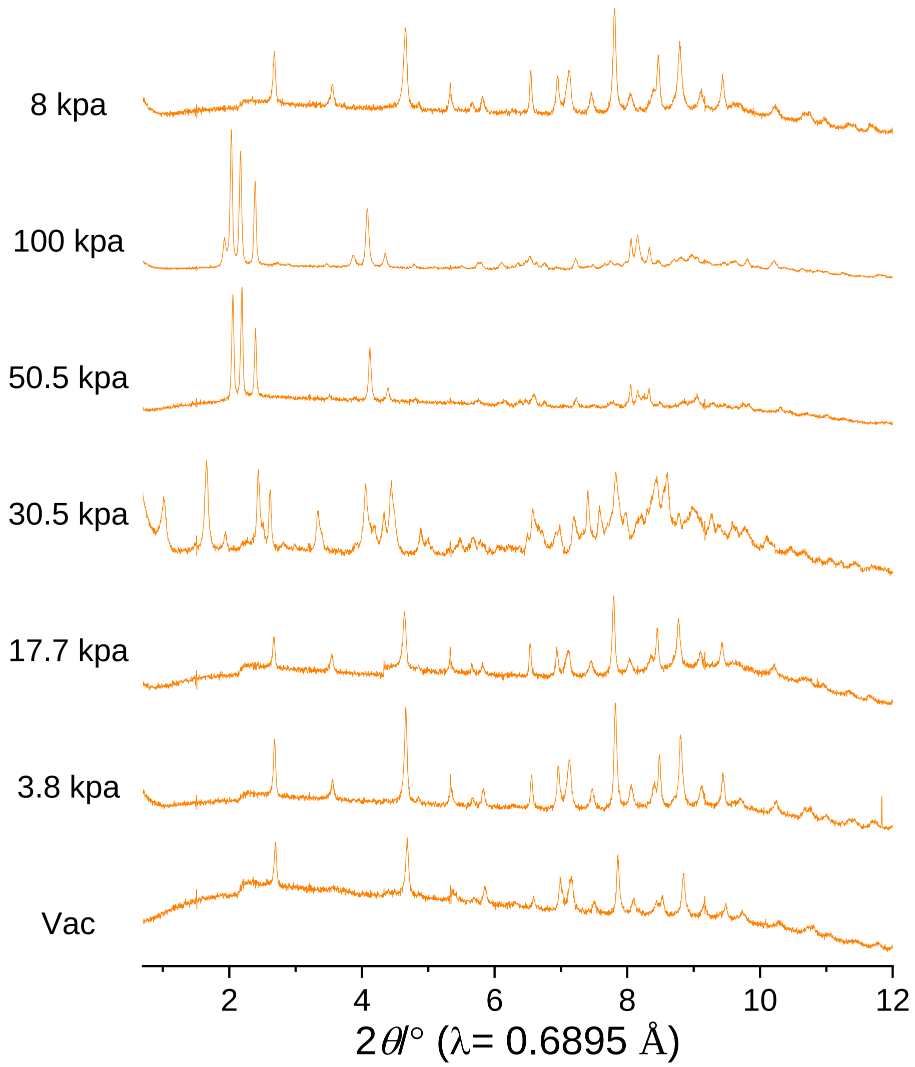

Supplementary Figure 33. PXRD patterns of dynaCOF-330 were obtained at the corresponding *n*-butane gas pressure. Three crystalline stages were observed for dynaCOF-330 during *n*-butane uptake at room temperature below 100 kPa. This crystal structure evolution is similar to the procedure of acetone inclusion. At 100 kPa, dynaCOF-330 is starting to enter stage IV but is not fully transformed.

Supplementary Table 3. The crystallography data of dynaCOF-330 was determined by synchrotron PXRD analyses.

**Samples**	**dynaCOF-330**	**dynaCOF-330I (acetone)**	**dynaCOF-330III (acetone)**	**dynaCOF-330IV (acetone)**	**dynaCOF-330I (dioxane)**	**dynaCOF-330III (dioxane)**	**dynaCOF-330IV (dioxane)**
**Beamline**	SSRF 14B1						
**Radiation**	Synchrotron X-ray						
**λ (Å)**	0.6895						
**T (K)**	298						
**Formula**	$C_{178}H_{136}N_8O_{16}$	$C_{184}H_{156}N_8O_{18}$	$C_{226}H_{232}N_8O_{32}$	$C_{238}H_{256}N_8O_{36}$	$C_{185.04}H_{150.08}N_8O_{19.52}$	$C_{226}H_{232}N_8O_{40}$	$C_{241.82}H_{136}N_8O_{47.9}$
**Guest/cell**	0	2	16	18.72	1.75	12	16
**Z**	4	4	4	8	4	8	8
**Crystal system**	Orthorhombic	Orthorhombic	Monoclinic	Tetragonal	Orthorhombic	Tetragonal	Tetragonal
**Space group**	$Pnn2$	$Pnn2$	$P2/n$	$P4_2/n$	$Pnn2$	$P4_2/n$	$P4_2/n$
**a (Å)**	16.83	16.95	22.17	28.80	16.95	24.96	27.84
**b (Å)**	29.11	29.22	7.32	28.80	29.25	24.96	27.84
**c (Å)**	7.64	7.56	32.50	7.20	7.67	8.00	7.26
**β (°)**	90	90	92.92	90	90	90	90
**V (Å^3)**	3743.00	3744.51	5271.37	5972.98	3803.37	4977.10	5624.13
**D_c (g cm^{-3})**	1.17	1.23	1.23	1.06	1.22	1.23	1.21
**2θ range (°)**	1.0-25						
**Method**	Rietveld	Pawley	Rietveld	Rietveld	Rietveld	Rietveld	Rietveld
**R_{wp} (%)**	5.896	5.27	4.544	1.976	4.325	7.576	7.861
**R_p (%)**	1.297	7.89	1.626	1.458	1.888	5.245	5.476

Supplementary Section 4. Fluorescence sensing of organic gas/vapours.

The organic vapor adsorption isotherms were collected using the MicrotracBELSorp-Aqua3 and BELSORP-max adsorption apparatus with a water circulator bath. Ultrahigh grade gases such as He and N_2 were used throughout the experiments. Anhydrous solvents were used for vapor adsorption and degassed for at least five times before isotherm collection.

***In-situ* fluorescence spectroscopy of single-component vapour sensing.** A special sample cell was prepared as shown in Supplementary Figure 34. DynaCOF-330 (about 40 mg) was filled in one side of the cell to avoid scattering during the fluorescent experiment. Then the sample-filled cell was placed in a quartz adsorption cell with an inner diameter of 9.0 mm. Before fluorescence spectrum measurement, the absorption cell was then treated in a vacuum at 100 °C for 24 hours to ensure the guest was entirely removed. No thermostat was used during organic vapour uptake, but the temperature was monitored with a thermometer during the measurement showing the temperature fluctuated less than 1 °C. Organic vapours dosing and pressure controlling were performed by BELSORP-max, which was connected to a photoluminescence spectrometer to realize *in-situ* fluorescence experiments. Equilibration time of 1800 – 3600 s at each equilibrium pressure was adopted to ensure the equilibrium conditions of 1% pressure change within 300 s.

During the organic vapour adsorption/desorption cycling experiments, fluorescent intensity is monitored. The sample tested for the first round was prepared by vacuuming at 373 K. After guests were fully removed, the sample was transferred to *in situ* devices to monitor the fluorescence intensity under a vacuum state with pressure < 3 Pa. Then acetone or 1,4-dioxane vapour was added to the sample to monitor the fluorescence intensity at chosen pressure equilibrium (maintained pressure changes <1% during 300 s). Since acetone molecules adsorbed in the channels of dynaCOF-330 can be easily removed, vacuuming the sample at room temperature (P < 3 Pa) yields a guest-free sample for the second-round test. This procedure was repeated 6 times to confirm reversible fluorescence changes. To fully remove the adsorbed 1,4-dioxane molecules in the COF channels an elongated vacuum duration or heating is needed.

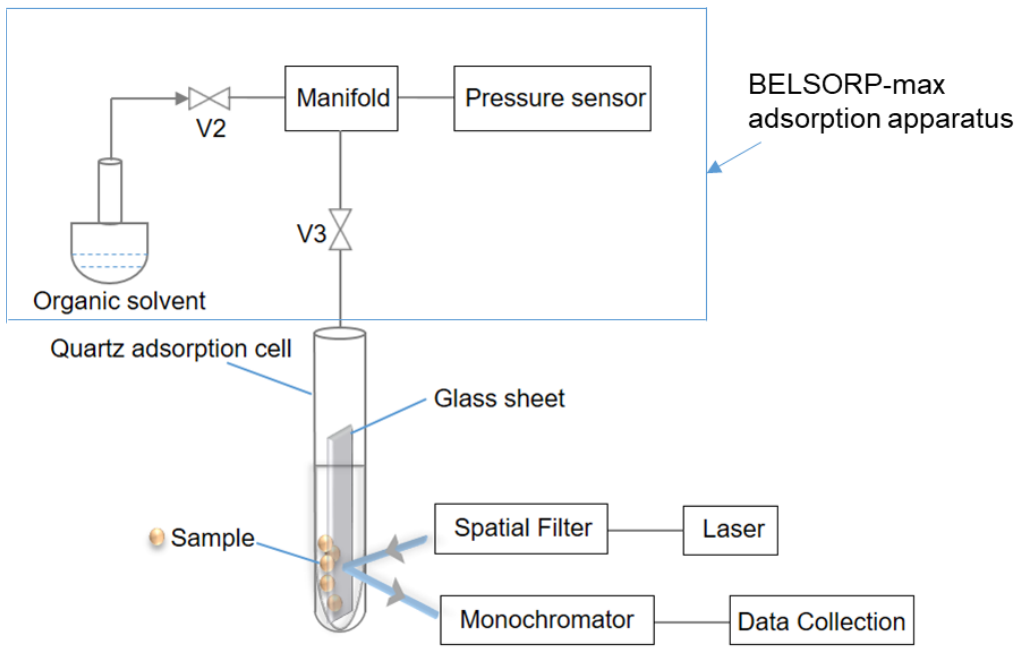

Supplementary Figure 34. Schematic illustration of the BELSORP-max connected to a photoluminescence spectrometer.

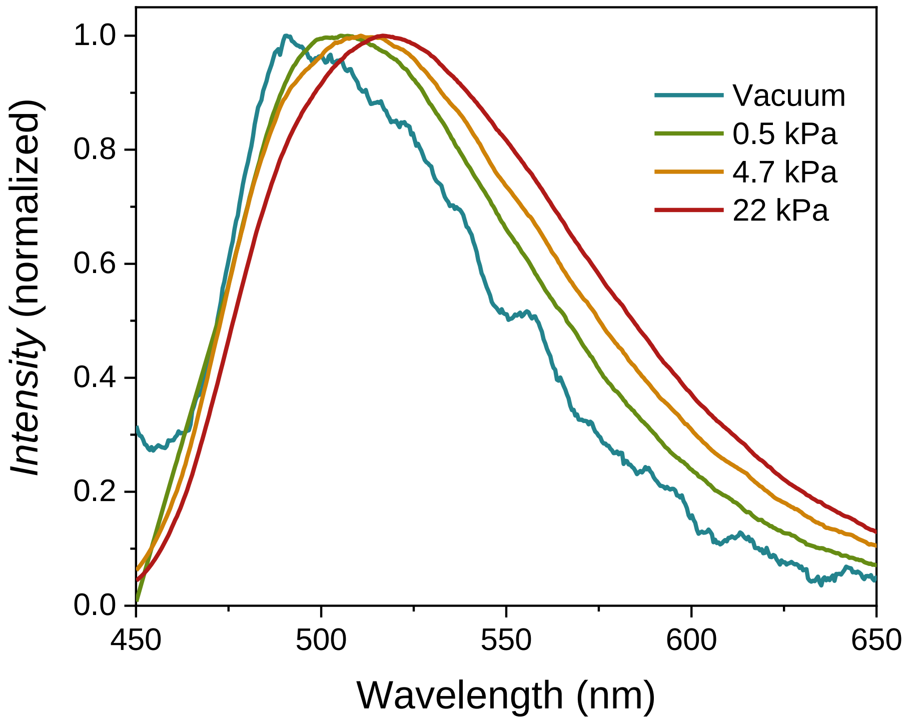

Supplementary Figure 35. The normalized fluorescent spectra of dynaCOF-330 under selected acetone vapour pressure. The maxima emission wavelength redshifts with increasing acetone uptake. The excitation wavelength is 420 nm. The maxima emission wavelength of dynaCOF-330 is maintained at 515 nm at the acetone vapour pressure above 22 kPa.

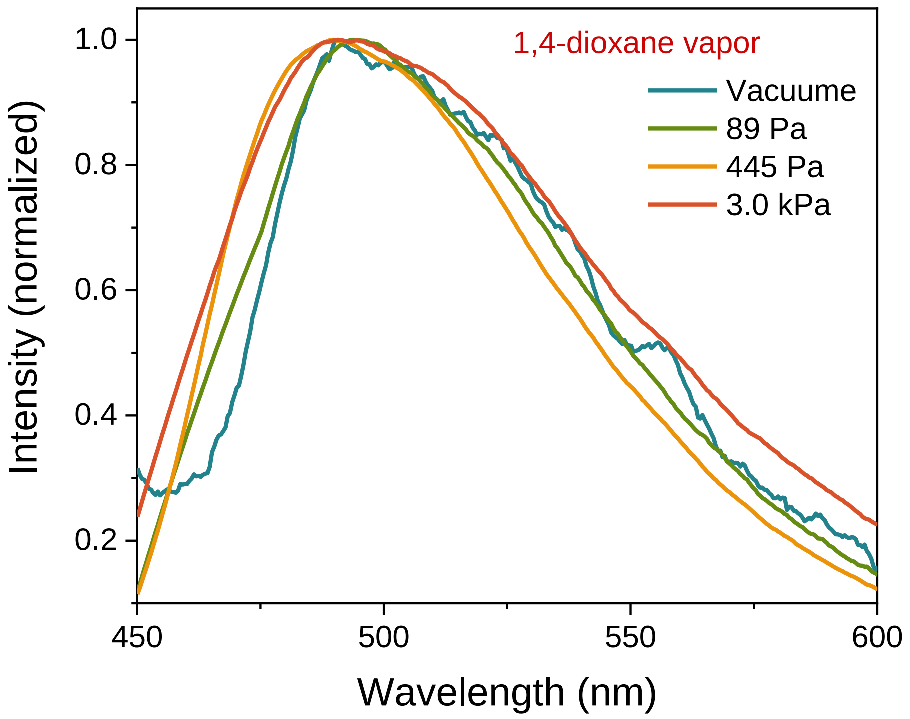

Supplementary Figure 36. The normalized fluorescent spectra of dynaCOF-330 at selected 1,4-dioxane vapour pressure. The emission maxima wavelength is slightly blue-shifted at a pressure range of 0.089-0.445 kPa. The excitation wavelength is 420 nm. As the polarizability of fluorophores in the presence/absence of 1,4-dioxane are similar, the maxima emission wavelength was almost unchanged during 1,4-dioxane uptake.

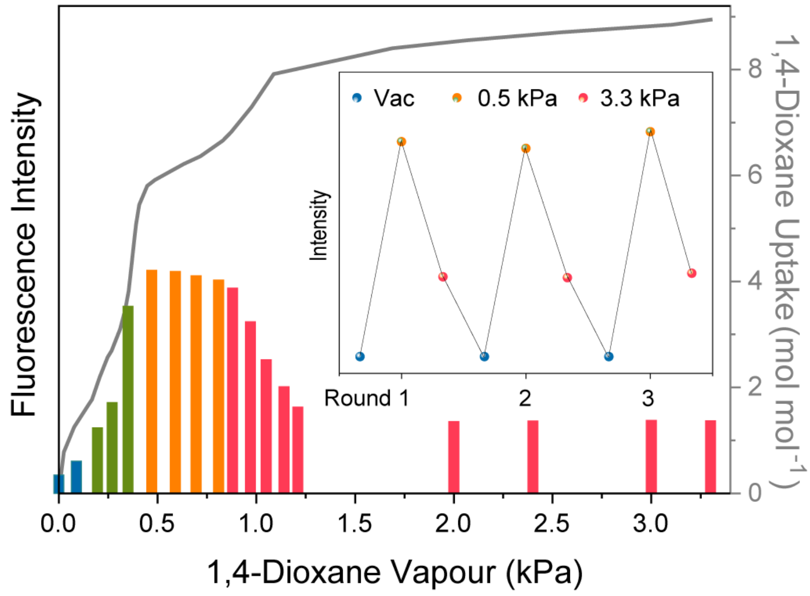

Supplementary Figure 37. Inverse correlation of fluorescence emission intensity with 1,4-dioxane vapour uptakes with an inset for the steady 1,4-dioxane vapour sensing through adaptive inclusion. At pressure lower than 0.45 kPa, the fluorescent enhanced with the pressure increment, but fluorescence quenched in the pressure range of 0.445-1.3 kPa.

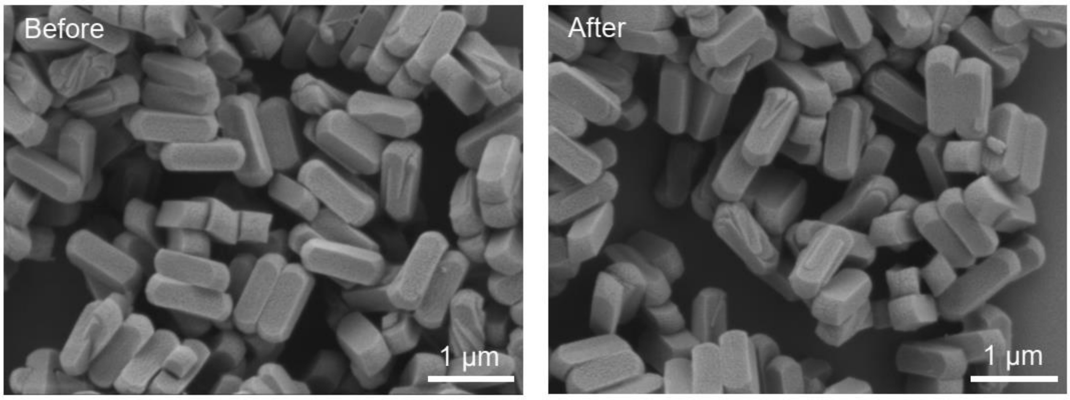

Supplementary Figure 38. SEM images of dynaCOF-330 after ten cycles of 1,4-dioxane vapour adsorption.

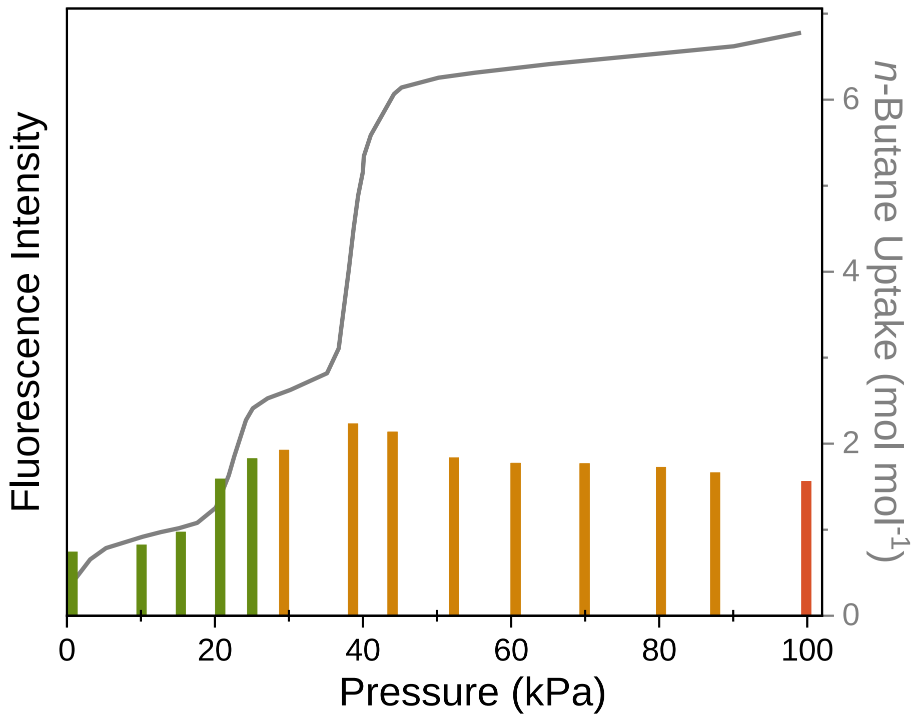

Supplementary Figure 39. Correlation of fluorescence emission intensity of dynaCOF-330 with *n*-butane uptakes.

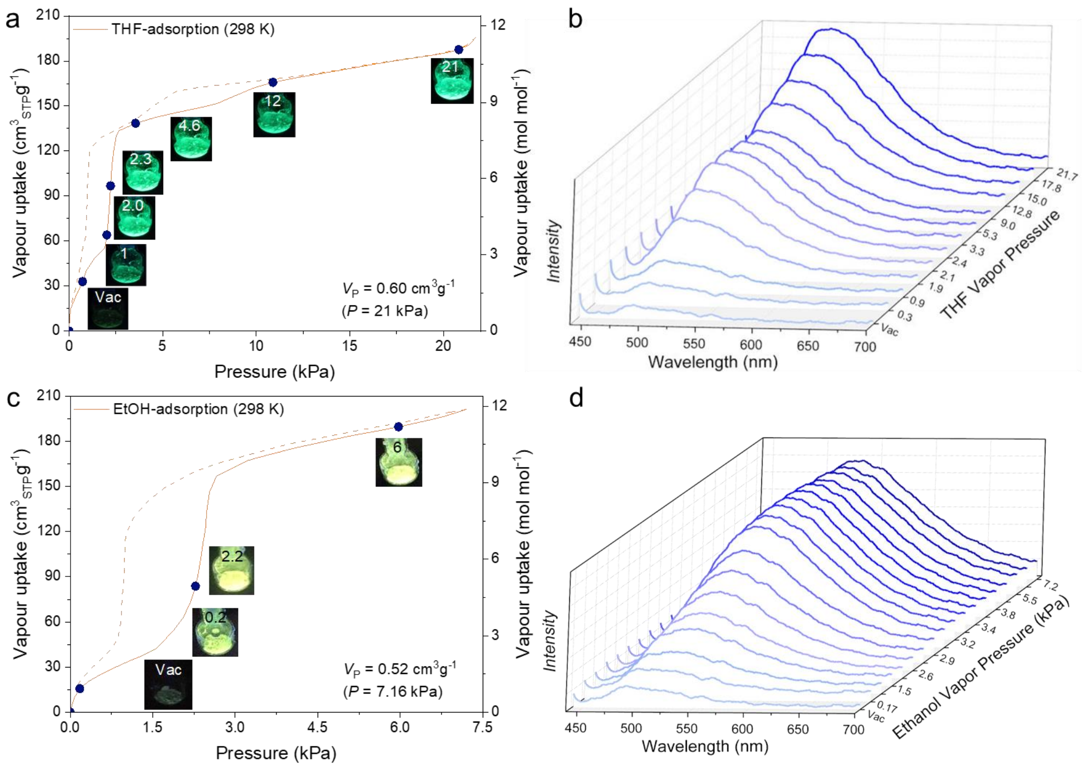

Supplementary Figure 40. Optical images of dynaCOF-330 under 365-nmUV lamp irradiation showing the fluorescence turn-on and intensity changes for representative equilibrium pressures and uptake of THF (a) and EtOH (c) vapour at 298 K. Quantitative *in-situ* fluorescence spectra of dynaCOF-300 during THF (b) and EtOH (d) vapour absorption process showing maxima redshift of emission wavelength. The excitation wavelength is 420 nm.

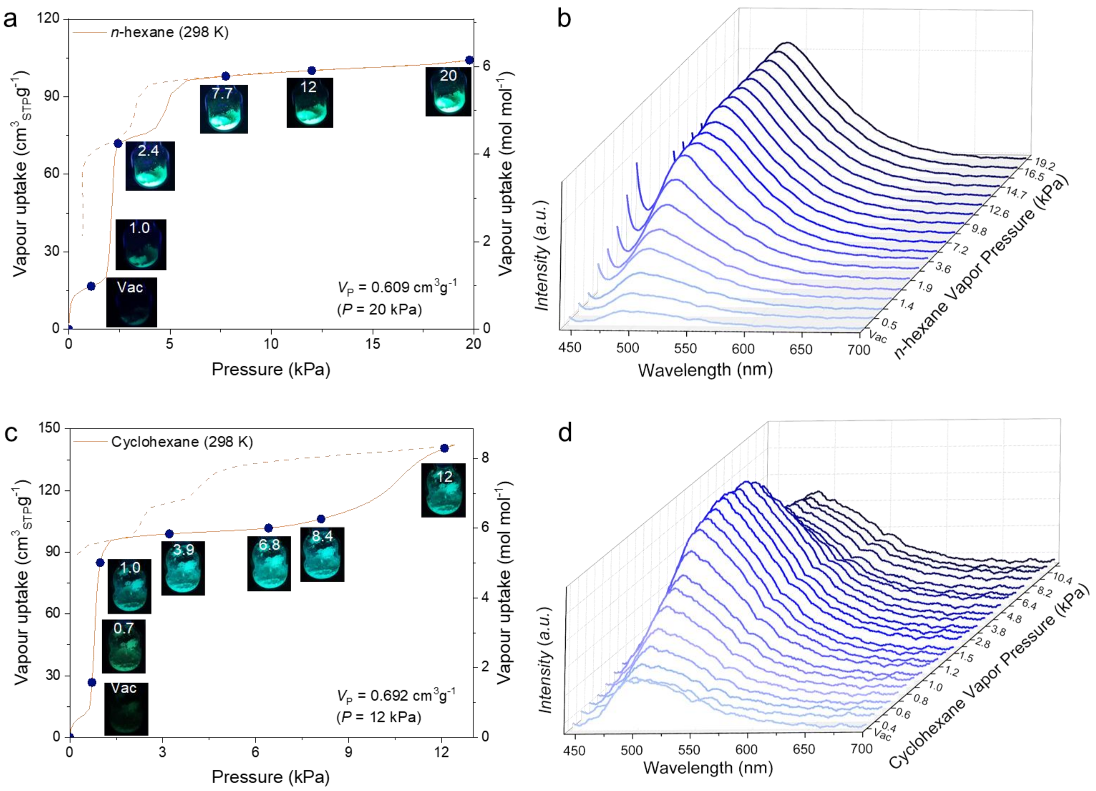

Supplementary Figure 41. Optical images of dynaCOF-330 under 365-nm UV lamp irradiation showing the fluorescence turn-on and intensity changes for representative equilibrium pressures and uptake of *n*-hexane (a) and cyclohexane (c) vapour at 298 K. Quantitative *in-situ* fluorescence spectra of dynaCOF-300 during n-hexane (b) and cyclohexane (d) vapour absorption showing maxima blueshift of emission wavelength. The irradiation wavelength is 400 nm.

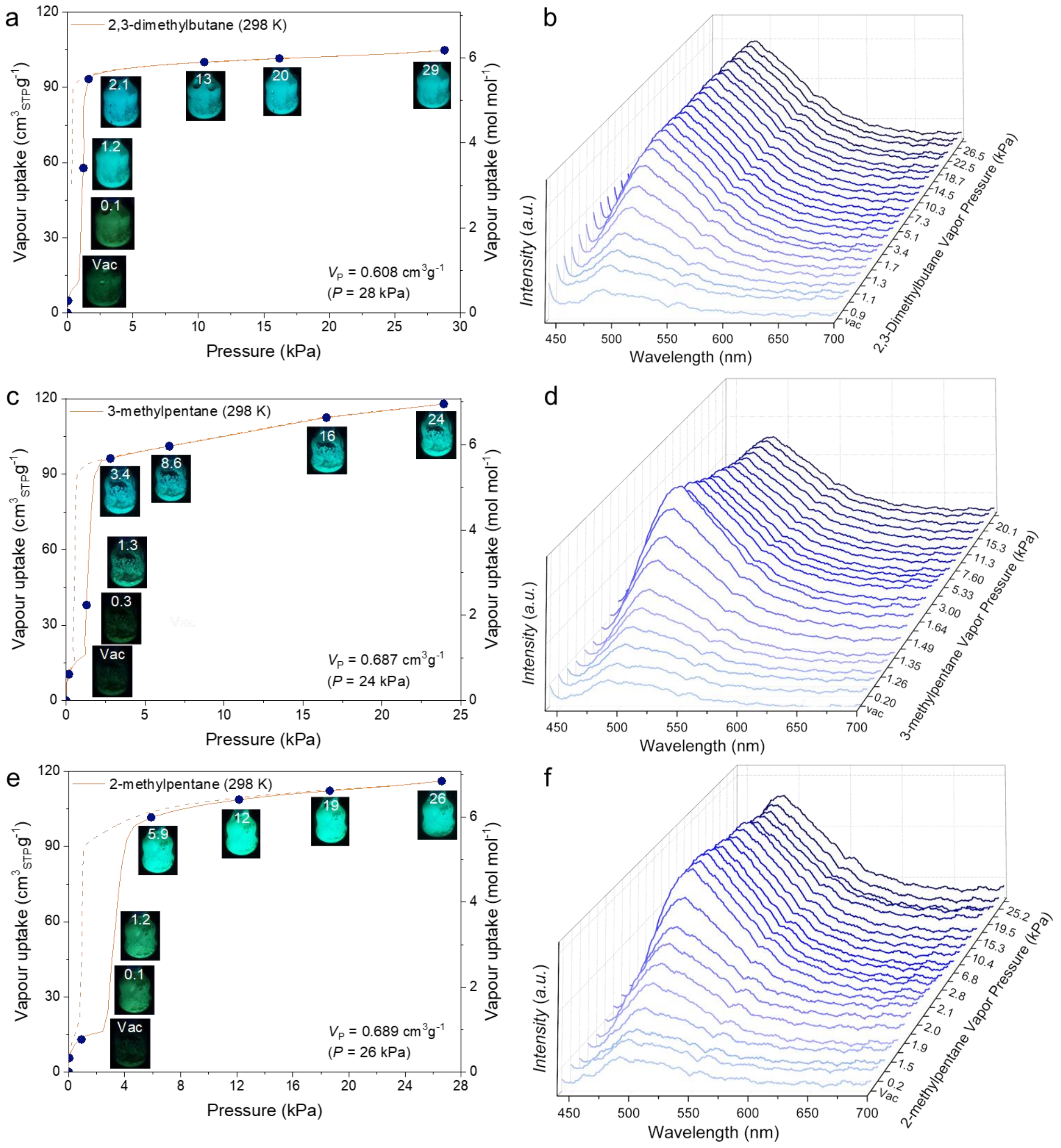

Supplementary Figure 42. Optical images of dynaCOF-330 under 365-nm UV lamp irradiation showing the fluorescence turn-on and intensity changes for representative equilibrium pressures and uptake of 2,3-dimethylbutane (a), 3-methylpentane (c), and 2-methylpentane (e) vapour at 298 K. Quantitative *in-situ* fluorescence spectra of dynaCOF-300 during 2,3-dimethylbutane (b), 3-methylpentane (d), and 2-methylpentane (f) vapour absorption showing maxima blueshift of emission wavelength. The irradiation wavelength is 400 nm. The fluorescence responses of dynaCOF-330 displayed shape selectivity for saturated C6 hydrocarbon isomers. For linear *n*-hexane, the fluorescence intensity of dynaCOF-330 enhanced with increasing vapour pressure and attained the highest emission intensity at 7.2 kPa. Cyclohexane as

the analyte, the highest emission intensity attained at 2.8 kPa. Further increases in organic vapour pressure fluorescence were quenched (Supplementary Figure 41). And the branched C6 isomers can be distinguished by the fluorescent response tendency of dynaCOF-330 during organic vapour uptake. The fluorescence response of 2,3-dimethylbutane is similar to *n*-hexane. DynaCOF-330 shows a similar fluorescence response for 2-methylpropane and 3-methylpropane, but the fluorescence quenching degree of 3-methylpropane is intensified.

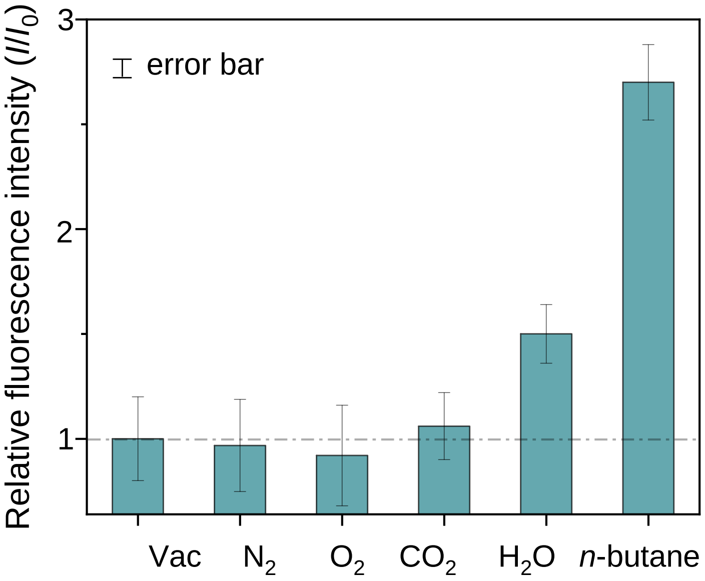

Supplementary Figure 43. Fluorescence response of dynaCOF-330 for ambient air compositions and *n*-butane.

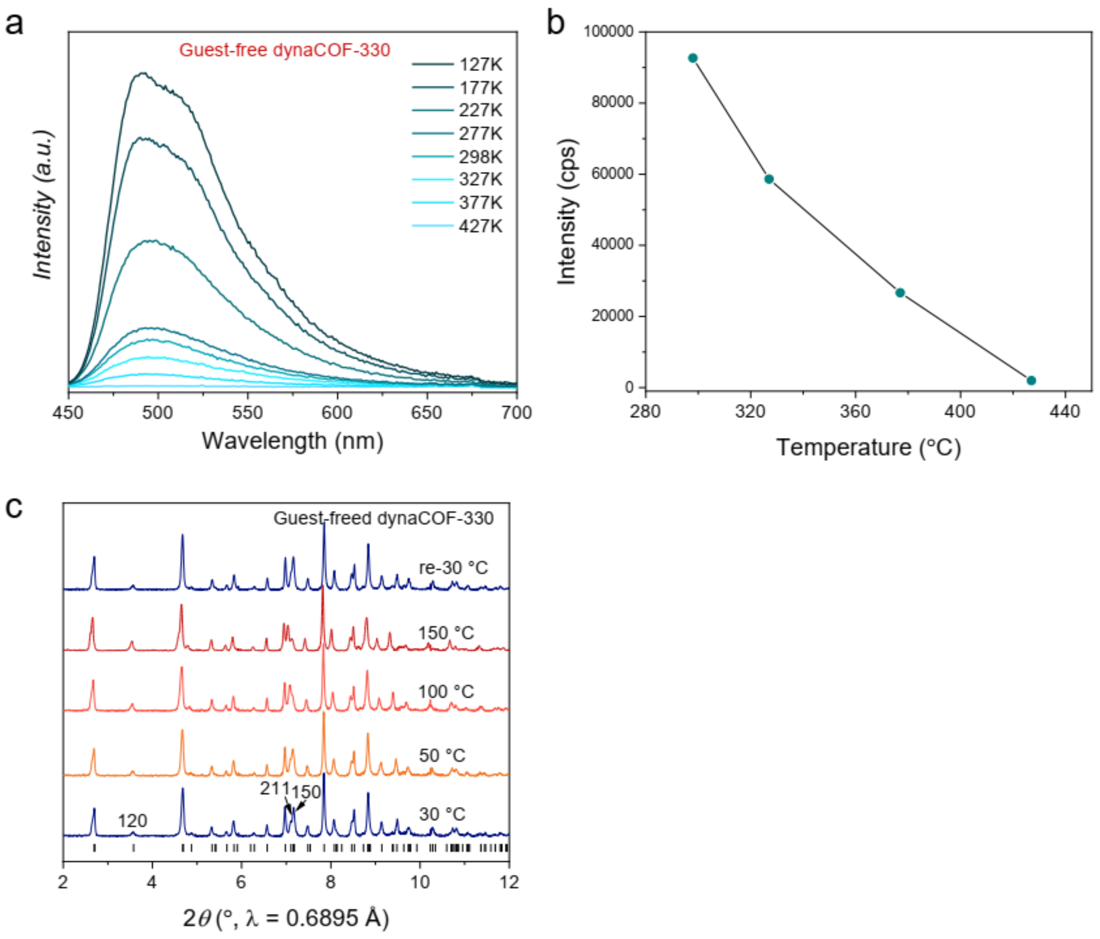

Supplementary Figure 44. (a) Temperature-dependent fluorescence spectra of guest-free dynaCOF-330; (b) Temperature-dependent fluorescence peak intensity and (c) PXRD patterns of guest-free dynaCOF-330 under different temperatures. Fluorescence quenching was observed with elevated temperature. From room temperature to 427 K the fluorescence intensity displays a negative linear correlation with temperature (b). The *in-situ* PXRD under varied temperatures illustrates the structure changed slightly upon temperature variation.

Supplementary Section 5. *In-situ* fluorescence spectroscopy for dynamic multi-component gas sensing.

A special customized relative humidity and gas partial pressure controller was prepared as shown in Supplementary Figure 45. Two mass flow controllers (MFCs) with different controlling ranges (100 sccm, and 5 sccm for low partial pressure less than 5%) were connected to *n*-C_4H_{10} cylinder. Two MFCs were connected to N_2 cylinder as a purge gas to activate the sample or balanced gas mixed with *n*-C_4H_{10}. The N_2 and *n*-C_4H_{10} mixture was then passed through a water bottle with saturated $MgCl_2$ to humidify the working gas with 53% relative humidity. A tee valve was used to switch the wet working gas to dry gas. All the stainless-steel valves and joints were purchased from Shanghai X-tec Fluid Technology Co, Ltd, and the MFCs were purchased from Alicat Scientific (A Halma company). The film sample was prepared by dropping a slurry of dynaCOF-330 (10 mg dispersed in 3 mL acetone) in the washed glass slide (1 cm × 3 cm) to measure its fluorescence response for *n*-butane gas. Before measurement, the COF film was dried under vacuum at room temperature for 5 hours to ensure the guest was entirely removed.

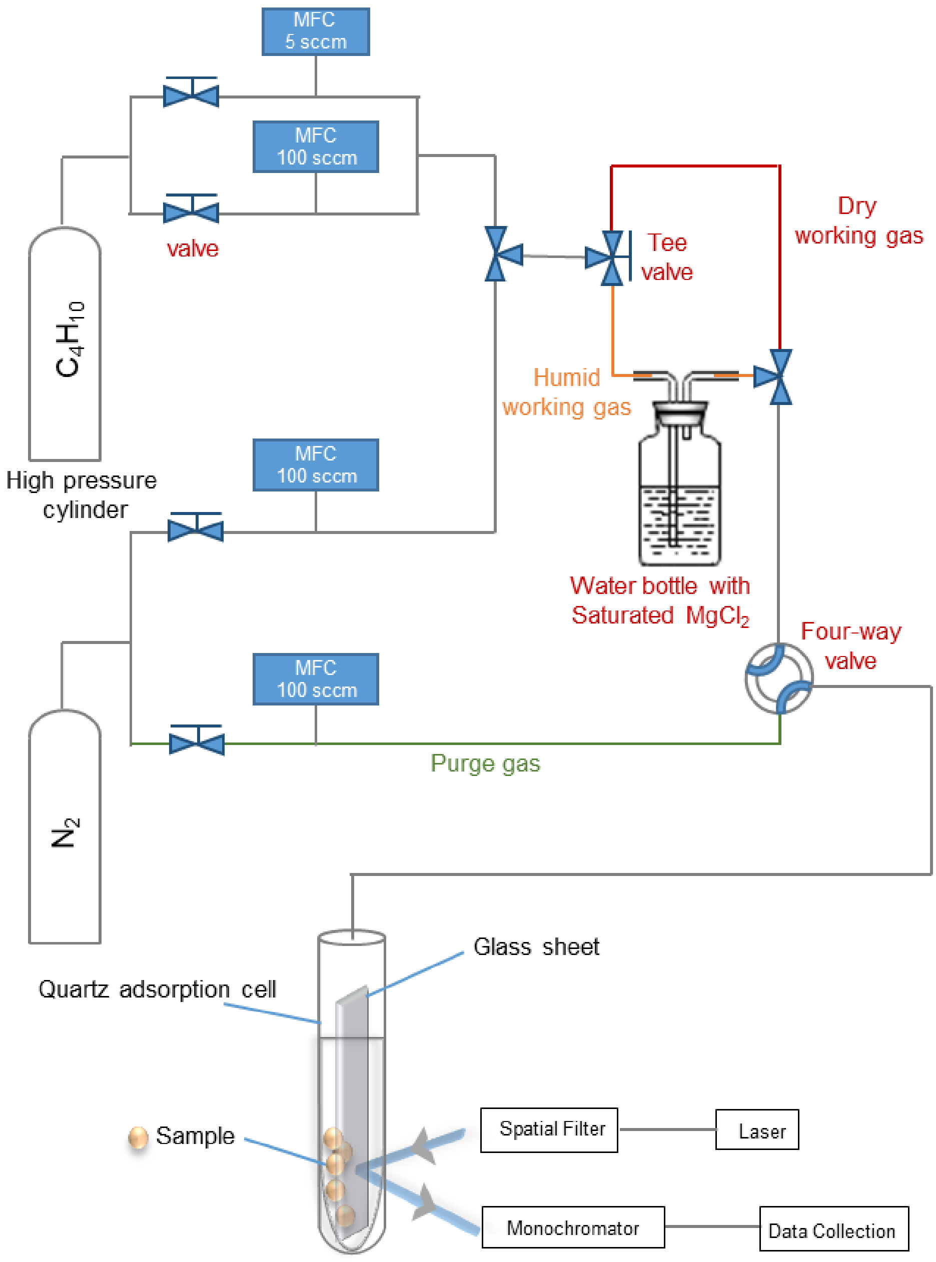

Supplementary Figure 45. Schematic illustration of the customized relative humidity and gas partial pressure controller connected to a photoluminescence spectrometer.

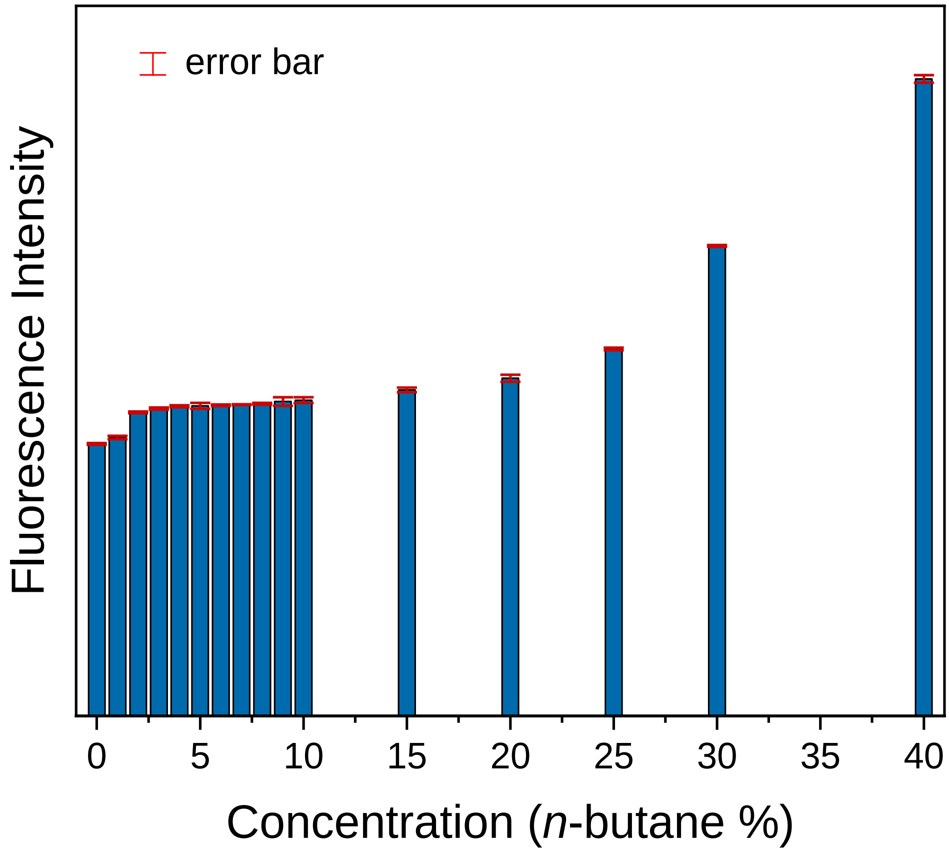

Supplementary Figure 46. Fluorescent intensity responses of dynaCOF-330 under varying concentrations of *n*-butane with dry N_2 as carrier gas. The *n*-butane response limitation of 2% corresponds to 10% increment in the fluorescence intensity of dynaCOF-330

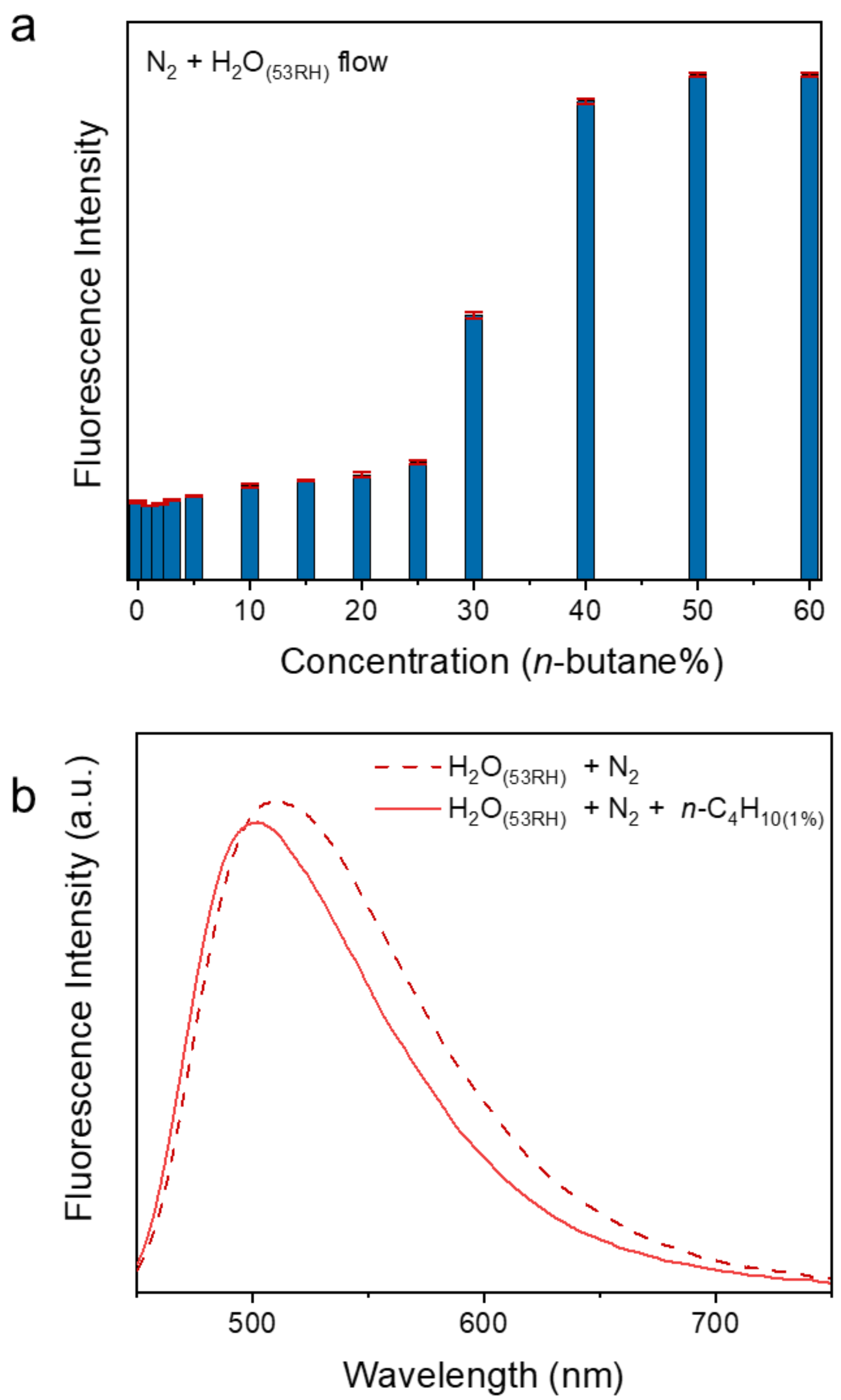

Supplementary Figure 47. Fluorescence responses of dynaCOF-330 under varying concentrations of *n*-butane carried by N_2 gas with 53% RH (a). The *n*-butane response limitation of 1% corresponds to 10-nm blue shifts to the maxima emission wavelength (b).

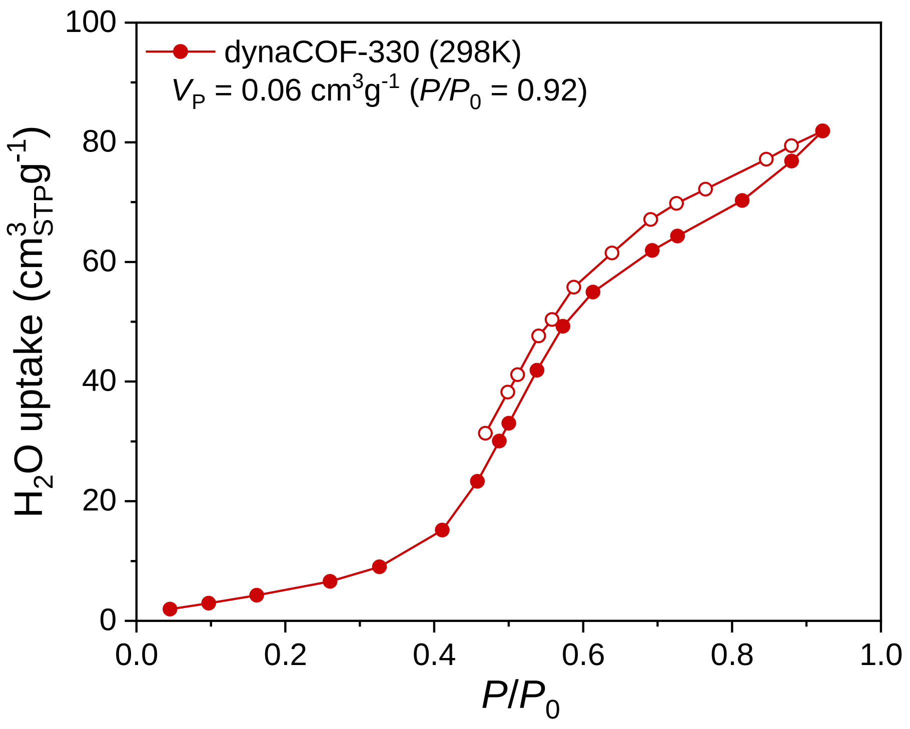

Supplementary Figure 48. The H_2O uptake of dynaCOF-330 at 195 K.

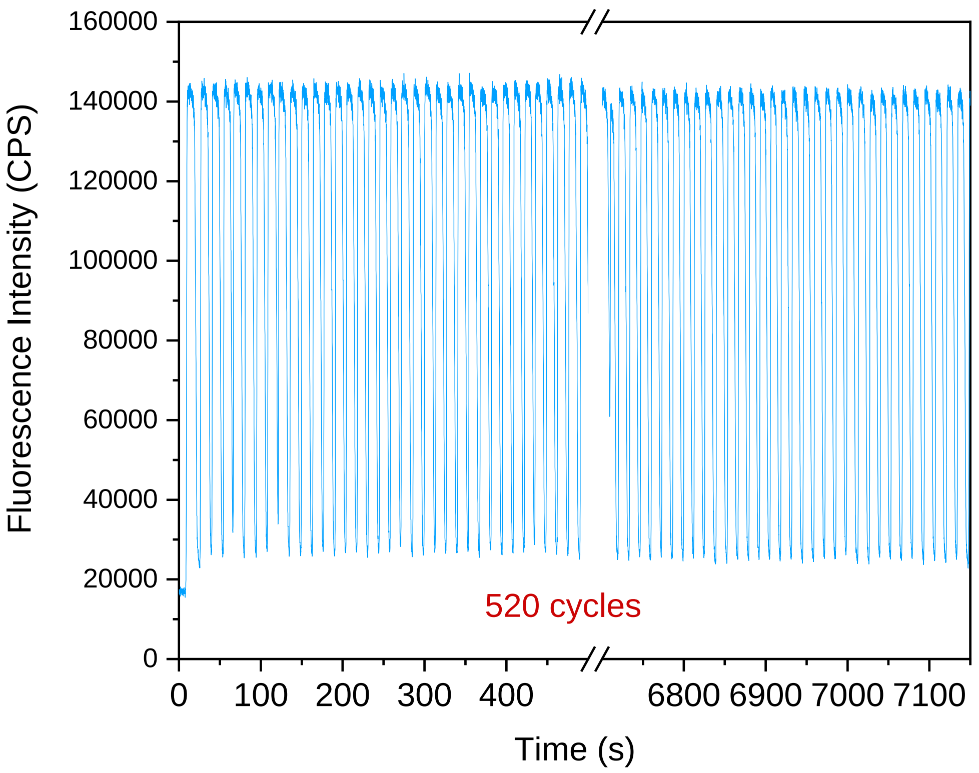

Supplementary Figure 49. The fluorescence response of dynaCOF-330 for dry 50% *n*-butane. The performance was stable after over five hundred response cycles.

Supplementary Section 6. Computational simulation

The simulation was performed by the Large-scale Atomic/Molecular Massively Parallel Simulator (LAMMPS) [8] code. The periodic boundary conditions were always used because the dynaCOF-330 is a kind of three-dimension extended framework. The force field used for dynaCOF-330 is OPLS_2005[9], and the parameters of the guest molecules (acetone) are assigned by the LigParGen [10] code. The supercells (2 × 1 × 4 unit cells) of the frameworks were used which contain sixteen 1,4-dioxane molecules. Here we chose a nano cell (2 × 1 × 4 unit cell) as the initial configuration for a 14 Å pairwise Lennard-Jones interactions and long-range Coulombic interactions with a cutoff of 10 Å. An additional damping factor is applied to the Coulombic term, which is the standard Ewald summation with an accuracy of 0.000001.

The equations of motion were updated through the Verlet leapfrog algorithm[11] and a time step of 1 fs. The system was first equilibrated for 500ps, followed by a 1500 ps production run. The simulation was carried out in an NPT ensemble, with a constant temperature of 298 K and a constant pressure of 0.1 MPa with all 3 diagonal components together when the pressure is computed. To control the temperature and pressure, a Nose-Hoover chains method [12] was applied during the whole process. For the result, the dihedral analysis was based on the production run. We chose the frame structure at 0.5 ps as the first initial frame. The first plane is composed of the three carbon atoms. Every 0.5 ps, we chose a shot from the trajectory and the second plane consists of the same three atoms. The angle between the two planes is the dihedral. When the dihedral is beyond 90 degrees, it means the whole fragment is upside down. For a better understanding of the flexibility of the COF, we rule the dihedral is 180 degrees minus angle when the dihedral is beyond 90 degrees.

Supplementary Section 7. Tables of fractional atomic coordinates

Supplementary Table 4. Fractional atomic coordinates for dynaCOF-330.

dynaCOF-330<br>Space group: *Pnn*2 (no.34); a = 16.83 Å; b = 29.11 Å; c = 7.64 Å;<br>$\alpha = \beta = \gamma = 90°$; V = 3743.00 Å^3; Z = 4					
**Atom**	***x***	***y***	***z***	**Occupancy**	**B_{iso} (Å^2)**
C1	0.0000	0.0000	0.0000	1.0000	1.0000
C10	0.4173	0.5626	0.3558	1.0000	4.3009
C11	0.5412	0.5904	0.1453	1.0000	4.3009
C12	0.4071	0.5982	0.2377	1.0000	4.3009
C13	0.4686	0.6121	0.1314	1.0000	4.3009
C14	0.9866	0.8394	0.3706	1.0000	4.3009
C15	0.9624	0.8088	0.2423	1.0000	4.3009
C16	0.9933	0.8145	0.0756	1.0000	4.3009
C17	0.9359	0.7659	0.2887	1.0000	4.3009
C18	0.9894	0.7799	-0.0486	1.0000	4.3009
C19	0.9334	0.7310	0.1661	1.0000	4.3009
C2	0.5736	0.5029	0.6178	1.0000	4.3009
C20	0.9546	0.7385	-0.0066	1.0000	4.3009
C21	0.9445	0.7051	-0.1228	1.0000	4.3009
C22	0.8705	0.7075	-0.2084	1.0000	4.3009
C23	0.8130	0.7383	-0.1509	1.0000	4.3009
C24	0.7407	0.7425	-0.2357	1.0000	4.3009
C25	0.7210	0.7117	-0.3634	1.0000	4.3009
C26	0.6726	0.7704	0.0174	1.0000	4.3009
C27	0.6362	0.7067	-0.6114	1.0000	4.3009
C28	0.7771	0.6809	-0.4224	1.0000	4.3009
C29	0.8521	0.6791	-0.3466	1.0000	4.3009
C3	0.5931	0.4675	0.7296	1.0000	4.3009
C30	0.9069	0.6475	-0.4051	1.0000	4.3009
C31	0.9837	0.6470	-0.3337	1.0000	4.3009
C32	1.0035	0.6763	-0.1942	1.0000	4.3009
C33	1.0407	0.6172	-0.3989	1.0000	4.3009
C34	1.0784	0.6730	-0.1188	1.0000	4.3009
C35	1.1171	0.6172	-0.3320	1.0000	4.3009
C36	1.1352	0.6443	-0.1898	1.0000	4.3009
C37	1.2206	0.6604	0.0496	1.0000	4.3009
C38	1.2082	0.5601	-0.2377	1.0000	4.3009
C39	0.8853	0.6213	-0.5463	1.0000	4.3009
C4	0.6122	0.5446	0.6348	1.0000	4.3009

C40	0.9310	0.6225	-0.6966	1.0000	4.3009
C41	0.9138	0.5940	-0.8370	1.0000	4.3009
C42	0.8235	0.5901	-0.5350	1.0000	4.3009
C43	0.8065	0.5617	-0.6752	1.0000	4.3009
C44	0.8509	0.5638	-0.8277	1.0000	4.3009
C45	0.8292	0.5365	-0.9690	1.0000	4.3009
C5	0.6683	0.5514	0.7645	1.0000	4.3009
C6	0.6505	0.4740	0.8568	1.0000	4.3009
C7	0.6867	0.5164	0.8793	1.0000	4.3009
C8	0.4894	0.5402	0.3691	1.0000	4.3009
C9	0.5515	0.5547	0.2635	1.0000	4.3009
H10	0.3655	0.5518	0.4425	1.0000	2.0000
H11	0.5929	0.6017	0.0594	1.0000	2.0000
H12	0.3473	0.6164	0.2277	1.0000	2.0000
H14	0.4614	0.3605	-0.1814	1.0000	2.0000
H16	0.4775	0.3487	0.5418	1.0000	2.0000
H17	0.5841	0.2590	0.9287	1.0000	2.0000
H18	0.4853	0.2857	0.3148	1.0000	2.0000
H19	0.5867	0.1954	0.7099	1.0000	2.0000
H23	0.3249	0.7392	0.4685	1.0000	2.0000
H26a	0.8555	0.2369	0.5631	1.0000	2.0000
H26b	0.8674	0.3004	0.5549	1.0000	2.0000
H26c	0.7669	0.2748	0.5825	1.0000	2.0000
H27a	0.3398	0.3280	0.3449	1.0000	2.0000
H27b	0.3297	0.2647	0.3196	1.0000	2.0000
H27c	0.4295	0.2907	0.3538	1.0000	2.0000
H28	0.2615	0.8432	-0.0348	1.0000	2.0000
H3	0.5620	0.4329	0.7174	1.0000	2.0000
H33	0.5248	0.9077	-0.0087	1.0000	2.0000
H34	0.5933	0.8059	0.5026	1.0000	2.0000
H37a	0.7415	0.3074	0.0338	1.0000	2.0000
H37b	0.7474	0.3650	0.1388	1.0000	2.0000
H37c	0.8390	0.3300	0.1099	1.0000	2.0000
H38a	0.7252	0.4381	0.7369	1.0000	2.0000
H38b	0.8061	0.4218	0.8908	1.0000	2.0000
H38c	0.8111	0.4774	0.7708	1.0000	2.0000
H4	0.5980	0.5739	0.5411	1.0000	2.0000
H40	0.9835	0.6471	-0.7060	1.0000	2.0000
H41	0.9519	0.5955	-0.9603	1.0000	2.0000
H42	0.7865	0.5879	-0.4102	1.0000	2.0000
H43	0.7554	0.5361	-0.6660	1.0000	2.0000
H45	0.8829	0.5287	-1.0548	1.0000	2.0000

H5	0.6993	0.5861	0.7765	1.0000	2.0000
H6	0.6678	0.4439	0.9440	1.0000	2.0000
H9	0.6119	0.5372	0.2732	1.0000	2.0000
N1	0.2455	0.4810	0.9862	1.0000	2.0000
N2	0.4601	0.6535	0.0283	1.0000	2.0000
O1	0.6821	0.7694	-0.1643	1.0000	2.0000
O2	0.6455	0.7112	-0.4309	1.0000	2.0000
O3	1.1683	0.5813	-0.3724	1.0000	2.0000
O4	1.2085	0.6407	-0.1113	1.0000	2.0000

Supplementary Table 5. Fractional atomic coordinates for dynaCOF-330III (acetone).

dynaCOF-330III (acetone)					
Space group: *P*2/*n* (No.13); *a* = 22.17 Å; *b* = 7.32 Å; *c* = 32.50 Å; $\alpha = \gamma = 90°$; $\beta = 92.92°$; *V* = 5265.43 Å^3; Z = 4					
**Atom**	***x***	***y***	***z***	**Occupancy**	B_{iso} **(Å^2)**
C1	0.7500	0.6746	0.2500	1.0000	5.0000
C10	0.9102	0.5584	0.3902	1.0000	2.9254
C11	0.9349	0.7073	0.4119	1.0000	2.9254
C12	0.9480	0.6933	0.4540	1.0000	2.9254
C13	0.9338	0.5337	0.4748	1.0000	2.9254
C14	0.9093	0.3850	0.4532	1.0000	2.9254
C15	0.9751	0.8545	0.4777	1.0000	2.9254
C16	0.9406	0.9485	0.5057	1.0000	2.9254
C17	0.8836	0.8845	0.5143	1.0000	2.9254
C18	0.8493	0.9771	0.5426	1.0000	2.9254
C19	0.8751	0.1165	0.5663	1.0000	2.9254
C2	0.7862	0.7959	0.2803	1.0000	2.9254
C20	0.9313	0.1846	0.5564	1.0000	2.9254
C21	0.9671	0.0845	0.5303	1.0000	2.9254
C22	0.7755	0.7338	0.5428	1.0000	2.9254
C23	0.8625	0.3595	0.6158	1.0000	2.9254
C24	0.7073	0.5535	0.2739	1.0000	2.9254
C25	0.7186	0.5280	0.3158	1.0000	2.9254
C26	0.6833	0.4081	0.3377	1.0000	2.9254
C27	0.6410	0.2995	0.3170	1.0000	2.9254
C28	0.6308	0.3202	0.2749	1.0000	2.9254
C29	0.6663	0.4404	0.2529	1.0000	2.9254
C3	0.7553	0.9037	0.3079	1.0000	2.9254
C30	0.5826	0.0269	0.3264	1.0000	2.9254
C31	0.5504	0.9070	0.3565	1.0000	2.9254
C32	0.5209	0.9886	0.3887	1.0000	2.9254
C33	0.5053	0.8853	0.4227	1.0000	2.9254
C34	0.5197	0.7011	0.4247	1.0000	2.9254
C35	0.5474	0.6178	0.3919	1.0000	2.9254
C36	0.5631	0.7212	0.3580	1.0000	2.9254
C37	0.5098	0.5952	0.4638	1.0000	2.9254
C38	0.4679	0.4544	0.4639	1.0000	2.9254
C39	0.4370	0.4046	0.4275	1.0000	2.9254
C4	0.7864	0.0297	0.3328	1.0000	2.9254

C40	0.3992	0.2532	0.4271	1.0000	2.9254
C41	0.3892	0.1599	0.4631	1.0000	2.9254
C42	0.4182	0.2132	0.4997	1.0000	2.9254
C43	0.4583	0.3585	0.5001	1.0000	2.9254
C44	0.3734	0.2899	0.3540	1.0000	2.9254
C45	0.3472	-0.1052	0.4960	1.0000	2.9254
C46	-0.4812	-0.7269	0.1847	0.4318	1.0088
C47	-0.5086	-0.8711	0.1624	0.4318	1.0088
C48	-0.5646	-0.8453	0.1416	0.4318	1.0088
C49	0.3813	0.7734	0.2471	1.0000	1.0088
C5	0.8467	0.0666	0.3259	1.0000	2.9254
C50	0.4168	0.8687	0.2767	1.0000	1.0088
C51	0.3906	0.9393	0.3116	1.0000	1.0088
C52	0.2587	0.2439	0.3083	0.8494	9.9906
C53	0.2078	0.3375	0.2921	0.8494	9.9906
C54	0.1736	0.4442	0.3179	0.8494	9.9906
C55	0.8189	0.6711	0.0294	1.0000	1.0087
C56	0.7632	0.6644	0.0476	1.0000	1.0087
C57	0.7346	0.4960	0.0530	1.0000	1.0087
C6	0.8781	0.9570	0.2989	1.0000	2.9254
C7	0.8475	0.8241	0.2755	1.0000	2.9254
C8	0.8659	0.2401	0.3884	1.0000	2.9254
C9	0.8976	0.3968	0.4109	1.0000	2.9254
H10	0.9013	0.5684	0.3568	1.0000	2.0000
H11	0.9449	0.8345	0.3955	1.0000	2.0000
H13	0.9432	0.5232	0.5082	1.0000	2.0000
H14	0.8997	0.2575	0.4696	1.0000	2.0000
H17	0.8617	0.7832	0.4938	1.0000	2.0000
H20	0.9512	0.2981	0.5743	1.0000	2.0000
H22a	0.7660	0.7514	0.5096	1.0000	2.0000
H22b	0.8073	0.6199	0.5475	1.0000	2.0000
H22c	0.7332	0.7012	0.5579	1.0000	2.0000
H23a	0.8754	0.4775	0.5970	1.0000	2.0000
H23b	0.8290	0.4035	0.6379	1.0000	2.0000
H23c	0.9028	0.3088	0.6329	1.0000	2.0000
H25	0.7522	0.6135	0.3323	1.0000	2.0000
H26	0.6907	0.3936	0.3712	1.0000	2.0000
H28	0.5972	0.2345	0.2584	1.0000	2.9254
H29	0.6586	0.4557	0.2195	1.0000	2.0000
H3	0.7066	0.8831	0.3115	1.0000	2.0000
H30	0.5957	0.9713	0.2966	1.0000	2.0000
H32	0.5146	0.1376	0.3890	1.0000	2.0000

H33	0.4855	0.9528	0.4493	1.0000	2.0000
H35	0.5577	0.4709	0.3931	1.0000	2.0000
H36	0.5838	0.6547	0.3316	1.0000	2.0000
H39	0.4468	0.4743	0.3985	1.0000	2.0000
H4	0.7610	0.1212	0.3526	1.0000	2.0000
H42	0.4098	0.1391	0.5284	1.0000	2.0000
H44a	0.3435	0.2190	0.3315	1.0000	2.0000
H44b	0.3597	0.4341	0.3557	1.0000	2.0000
H44c	0.4200	0.2826	0.3437	1.0000	2.0000
H45a	0.3166	-0.2131	0.4843	1.0000	2.0000
H45b	0.3896	-0.1681	0.5073	1.0000	2.0000
H45c	0.3261	-0.0347	0.5216	1.0000	2.0000
H46a	-0.5173	-0.6267	0.1942	0.4318	2.0000
H46b	-0.4481	-0.6537	0.1646	0.4318	2.0000
H46c	-0.4559	-0.7830	0.2134	0.4318	2.0000
H48a	-0.5905	-0.9798	0.1405	0.4318	2.0000
H48b	-0.5576	-0.7965	0.1089	0.4318	2.0000
H48c	-0.5915	-0.7385	0.1585	0.4318	2.0000
H49a	0.3322	0.8186	0.2484	1.0000	2.0000
H49b	0.3847	0.6205	0.2535	1.0000	2.0000
H49c	0.3979	0.8034	0.2152	1.0000	2.0000
H51a	0.3932	0.8330	0.3372	1.0000	2.0000
H51b	0.3414	0.9748	0.3036	1.0000	2.0000
H51c	0.4160	0.0675	0.3223	1.0000	2.0000
H52a	0.2538	0.2185	0.3426	0.8494	2.0000
H52b	0.3008	0.3295	0.3039	0.8494	2.0000
H52c	0.2629	0.1077	0.2917	0.8494	2.0000
H54a	0.1245	0.4453	0.3056	0.8494	2.0000
H54b	0.1918	0.5897	0.3187	0.8494	2.0000
H54c	0.1765	0.3845	0.3503	0.8494	2.0000
H55a	0.8460	0.7924	0.0423	1.0000	2.0000
H55b	0.8113	0.6869	-0.0053	1.0000	2.0000
H55c	0.8448	0.5395	0.0365	1.0000	2.0000
H57a	0.7699	0.3825	0.0545	1.0000	2.0000
H57b	0.7008	0.4701	0.0260	1.0000	2.0000
H57c	0.7100	0.4984	0.0829	1.0000	2.0000
H6	0.9266	0.9796	0.2949	1.0000	2.0000
H7	0.8724	0.7384	0.2540	1.0000	2.0000
H8	0.8371	0.1483	0.4058	1.0000	2.0000
N1	0.8778	0.1981	0.3501	1.0000	9.9972
N2	0.6049	0.1825	0.3396	1.0000	9.9972
O1	0.7907	0.9171	0.5496	1.0000	5.1653

O2	0.8380	0.2182	0.5916	1.0000	5.1653
O3	0.3710	0.1895	0.3905	1.0000	5.1653
O4	0.3488	0.0137	0.4625	1.0000	5.1653
O5	-0.4802	-0.0252	0.1588	0.4318	1.0088
O6	0.4748	0.8811	0.2734	1.0000	1.0088
O7	0.1945	0.3353	0.2527	0.8494	9.9906
O8	0.7362	0.8154	0.0568	1.0000	1.0087

Supplementary Table 6. Fractional atomic coordinates for dynaCOF-330IV (acetone).

dynaCOF-330IV (acetone)					
Space group: $P4_2/n$ (No.86); $a = b$ = 28.80 Å; c = 7.20 Å; $\alpha = \beta = \gamma = 90^\circ$; V = 5972.96 Å^3; Z = 8					
**Atom**	***x***	***y***	***z***	**Occupancy**	B_{iso} **(Å^2)**
C1	0.7848	0.2282	0.3705	1.0000	9.9993
C10	0.8183	0.2052	0.4830	1.0000	0.1596
C11	0.8224	0.2174	0.6669	1.0000	0.1596
C12	0.8484	0.1709	0.4178	1.0000	0.1596
C13	0.8578	0.1994	0.7778	1.0000	0.1596
C14	0.8860	0.1553	0.5265	1.0000	0.1596
C15	0.8891	0.1675	0.7099	1.0000	0.1596
C16	0.9175	0.1398	0.8376	1.0000	0.1596
C17	0.0768	0.9175	0.1074	1.0000	10.0000
C18	0.0983	0.9335	0.2677	1.0000	10.0000
C19	0.0732	0.9591	0.3966	1.0000	10.0000
C2	0.7408	0.2051	0.3423	1.0000	9.9993
C20	0.0299	0.9268	0.0826	1.0000	10.0000
C21	0.0051	0.9506	0.2156	1.0000	10.0000
C22	0.0280	0.9716	0.3588	1.0000	10.0000
C23	0.0000	0.0000	0.5000	1.0000	10.0000
C24	0.8421	-0.4985	-0.5115	0.5100	9.4809
C25	0.8007	-0.4936	-0.6112	0.5100	9.4809
C26	0.8017	-0.4929	-0.8058	0.5100	9.4809
C27	0.5273	-0.7238	0.1641	0.6976	9.9948
C28	0.5245	-0.6778	0.1040	0.6976	9.9948
C29	0.5176	-0.6421	0.2332	0.6976	9.9948
C3	0.7066	0.2275	0.2218	1.0000	9.9993
C30	0.8907	-0.1720	0.0596	0.1287	1.0022
C31	0.9016	-0.2104	0.9490	0.1287	1.0022
C32	0.9024	-0.2549	0.0272	0.1287	1.0022
C33	0.0757	0.4862	0.0575	0.8011	10.0000
C34	0.0498	0.4457	0.0824	0.8011	10.0000
C35	0.0685	0.4085	0.1827	0.8011	10.0000
C4	0.6638	0.2009	0.1917	1.0000	9.9993
C5	0.6516	0.1630	0.2945	1.0000	9.9993
C6	0.6844	0.1421	0.4142	1.0000	9.9993
C7	0.7273	0.1632	0.4427	1.0000	9.9993
C8	0.9239	0.3355	0.3411	1.0000	9.9993
C9	0.6986	0.0726	0.6093	1.0000	9.9993

H11	0.8016	0.2475	0.7191	1.0000	3.0000
H12	0.8442	0.1571	0.2721	1.0000	3.0000
H13	0.8560	0.2048	0.9303	1.0000	3.0000
H14	0.9089	0.1266	0.4734	1.0000	3.0000
H16	0.9563	0.1396	0.8163	1.0000	3.0000
H18	0.1371	0.9308	0.2788	1.0000	1.0010
H19	0.0887	0.9671	0.5373	1.0000	1.0010
H20	1.0109	0.9115	0.9615	1.0000	1.0010
H21	0.9678	0.9581	0.1924	1.0000	1.0010
H24a	0.8347	-0.5171	-0.3748	0.5100	2.0000
H24b	0.8573	-0.4628	-0.4816	0.5100	2.0000
H24c	0.8679	-0.5197	-0.5966	0.5100	2.0000
H26a	0.8339	-0.5120	-0.8572	0.5100	2.0000
H26b	0.8025	-0.4555	-0.8565	0.5100	2.0000
H26c	0.7693	-0.5109	-0.8620	0.5100	2.0000
H27a	0.5508	-0.7443	0.0664	0.6976	2.0000
H27b	0.4912	-0.7399	0.1642	0.6976	2.0000
H27c	0.5422	-0.7249	0.3107	0.6976	2.0000
H29a	0.5307	-0.6537	0.3753	0.6976	2.0000
H29b	0.4791	-0.6336	0.2424	0.6976	2.0000
H29c	0.5376	-0.6099	0.1872	0.6976	2.0000
H30a	0.8731	-0.1444	0.9708	0.1287	2.0000
H30b	0.9239	-0.1572	0.1223	0.1287	2.0000
H30c	0.8662	-0.1832	0.1758	0.1287	2.0000
H32a	0.8791	-0.2557	0.1551	0.1287	2.0000
H32b	0.9394	-0.2640	0.0690	0.1287	2.0000
H32c	0.8893	-0.2812	0.9210	0.1287	2.0000
H33a	0.0512	0.5172	0.0469	0.8011	2.0000
H33b	0.0970	0.4833	0.9245	0.8011	2.0000
H33c	0.1000	0.4912	0.1809	0.8011	2.0000
H35a	0.0961	0.4220	0.2828	0.8011	2.0000
H35b	0.0850	0.3826	0.0824	0.8011	2.0000
H35c	0.0398	0.3905	0.2645	0.8011	2.0000
H4	0.8627	0.2853	0.4089	1.0000	1.0000
H7	0.7498	0.1510	0.5630	1.0000	1.0000
H8a	0.5712	0.1422	0.0313	1.0000	1.0000
H8b	0.5900	0.1990	0.1166	1.0000	1.0000
H8c	0.5416	0.1697	0.2292	1.0000	1.0000
H9a	0.7360	0.0819	0.5871	1.0000	1.0000
H9b	0.6939	0.0343	0.5889	1.0000	1.0000
H9c	0.6880	0.0823	0.7530	1.0000	1.0000
N1	0.1011	0.8895	0.9753	1.0000	0.1596

O1	0.6720	0.0956	0.4896	1.0000	9.9993
O2	0.8916	0.3572	0.2165	1.0000	9.9993
O3	0.7620	-0.4865	-0.5230	0.5100	9.4809
O4	0.5251	-0.6686	0.9272	0.6976	9.9948
O5	0.9139	-0.2042	0.7771	0.1287	1.0022
O6	0.0096	0.4415	0.0016	0.8011	10.0000

Supplementary Table 7 Fractional atomic coordinates for dynaCOF-330I (dioxane).

dynaCOF-330I (dioxane)					
Space group: *Pnn*2 (No.34); a = 16.95 Å; b = 29.25 Å; c = 7.67 Å; $\alpha = \beta = \gamma = 90^\circ$; V = 3803.375 Å^3; Z = 4					
**Atom**	***x***	***y***	***z***	**Occupancy**	B_{iso} (Å^2)
C1	0.0000	0.0000	0.0000	1.0000	1.0000
C10	0.5481	0.5533	0.2687	1.0000	8.2773
C11	0.5410	0.5903	0.1570	1.0000	8.2773
C12	0.4215	0.6067	0.2776	1.0000	8.2773
C13	0.4774	0.6180	0.1559	1.0000	8.2773
C14	0.9736	0.8432	0.3835	1.0000	8.2773
C15	0.9815	0.8065	0.2632	1.0000	8.2773
C16	1.0019	0.8122	0.0982	1.0000	8.2773
C17	0.9526	0.7657	0.2890	1.0000	8.2773
C18	0.9482	0.7330	0.1682	1.0000	8.2773
C19	0.9939	0.7790	-0.0283	1.0000	8.2773
C2	0.5736	0.5029	0.6178	1.0000	8.2773
C20	0.9643	0.7384	0.0077	1.0000	8.2773
C21	0.9445	0.7051	-0.1228	1.0000	8.2773
C22	0.8697	0.7099	-0.2134	1.0000	8.2773
C23	0.8081	0.7424	-0.1667	1.0000	8.2773
C24	0.7359	0.7427	-0.2569	1.0000	8.2773
C25	0.7247	0.7139	-0.3699	1.0000	8.2773
C26	0.7769	0.6844	-0.4320	1.0000	8.2773
C27	0.8530	0.6812	-0.3566	1.0000	8.2773
C28	0.9125	0.6486	-0.4064	1.0000	8.2773
C29	0.9829	0.6461	-0.3314	1.0000	8.2773
C3	0.5917	0.4686	0.7234	1.0000	8.2773
C30	1.0032	0.6750	-0.1943	1.0000	8.2773
C31	1.0477	0.6159	-0.3821	1.0000	8.2773
C32	1.1183	0.6142	-0.3262	1.0000	8.2773
C33	1.1408	0.6438	-0.2028	1.0000	8.2773
C34	1.0811	0.6683	-0.1179	1.0000	8.2773
C35	1.2322	0.6605	0.0106	1.0000	8.2773
C36	1.2123	0.5654	-0.2412	1.0000	8.2773
C37	0.8855	0.6230	-0.5523	1.0000	8.2773
C38	0.9397	0.6172	-0.6833	1.0000	8.2773
C39	0.9210	0.5911	-0.8315	1.0000	8.2773
C4	0.6466	0.4732	0.8589	1.0000	8.2773
C40	0.8477	0.5718	-0.8488	1.0000	8.2773

C41	0.7949	0.5752	-0.7124	1.0000	8.2773
C42	0.8131	0.6015	-0.5667	1.0000	8.2773
C43	0.8201	0.5494	-0.9804	1.0000	8.2773
C44	0.0898	0.5239	0.2542	0.4401	6.0000
C45	0.1650	0.5109	0.3555	0.4401	6.0000
C46	0.2187	0.5870	0.3085	0.4401	6.0000
C47	0.1442	0.6009	0.2066	0.4401	6.0000
C48	0.6321	0.6964	-0.5657	1.0000	8.2773
C49	0.6763	0.7684	-0.0295	1.0000	8.2773
C5	0.6089	0.5439	0.6304	1.0000	8.2773
C6	0.6641	0.5511	0.7597	1.0000	8.2773
C7	0.6867	0.5164	0.8793	1.0000	8.2773
C8	0.4924	0.5432	0.3829	1.0000	8.2773
C9	0.4287	0.5696	0.3869	1.0000	8.2773
H10	0.3994	0.4648	0.2481	1.0000	2.0000
H11	0.4129	0.4028	0.0584	1.0000	2.0000
H12	0.6382	0.3711	0.2732	1.0000	2.0000
H14	0.4518	0.6244	-0.2241	1.0000	2.0000
H16	0.4691	0.3492	0.5587	1.0000	2.0000
H17	0.5674	0.2586	0.9397	1.0000	2.0000
H18	0.5821	0.1977	0.7133	1.0000	2.0000
H19	0.4856	0.2883	0.3237	1.0000	2.0000
H23	0.3256	0.7332	0.4511	1.0000	2.0000
H26	0.2557	0.8487	-0.0052	1.0000	2.0000
H3	0.4379	0.5672	0.7170	1.0000	2.0000
H31	0.5267	0.9073	-0.0063	1.0000	2.0000
H34	0.5908	0.8058	0.5063	1.0000	2.0000
H35a	1.2624	0.6309	0.0815	1.0000	2.0000
H35b	1.2703	0.6925	0.0212	1.0000	2.0000
H35c	0.3280	0.1672	0.5724	1.0000	2.0000
H36a	0.7524	0.4066	0.8345	1.0000	2.0000
H36b	0.7373	0.4592	0.7004	1.0000	2.0000
H36c	0.8230	0.4555	0.8536	1.0000	2.0000
H38	0.9994	0.6348	-0.6695	1.0000	2.0000
H39	0.9638	0.5864	-0.9408	1.0000	2.0000
H4	0.3353	0.5550	0.9514	1.0000	2.0000
H41	0.7361	0.5593	-0.7292	1.0000	2.0000
H42	0.7704	0.6067	-0.4617	1.0000	2.0000
H43	0.8722	0.5349	-1.0718	1.0000	2.0000
H44a	0.0376	0.5037	0.3071	0.4401	2.0000
H44b	0.0981	0.5159	0.1099	0.4401	2.0000
H45a	0.1762	0.4727	0.3417	0.4401	2.0000

H45b	0.1568	0.5198	0.4992	0.4401	2.0000
H46a	0.2102	0.5950	0.4527	0.4401	2.0000
H46b	0.2718	0.6066	0.2566	0.4401	2.0000
H47a	0.1526	0.5926	0.0625	0.4401	2.0000
H47b	0.1331	0.6391	0.2231	0.4401	2.0000
H48a	0.6765	0.6679	-0.6303	1.0000	2.0000
H48b	0.6303	0.7236	-0.6927	1.0000	2.0000
H48c	0.4314	0.3224	0.4078	1.0000	2.0000
H49a	0.8542	0.2347	0.5032	1.0000	2.0000
H49b	0.7609	0.2680	0.5241	1.0000	2.0000
H49c	0.6421	0.7979	0.0321	1.0000	2.0000
H5	0.4034	0.4262	0.5439	1.0000	2.0000
H6	0.3029	0.4140	0.7809	1.0000	2.0000
H9	0.6261	0.4377	0.4814	1.0000	2.0000
N1	0.2553	0.4721	1.0076	1.0000	3.4331
N2	0.4691	0.6516	0.0689	1.0000	3.4331
O1	0.6786	0.7743	-0.2143	1.0000	2.0000
O2	0.6378	0.6977	-0.3795	1.0000	2.0000
O3	1.1800	0.5862	-0.3875	1.0000	2.0000
O4	1.2221	0.6489	-0.1671	1.0000	2.0000
O5	0.2323	0.5363	0.2857	0.4401	6.0000
O6	0.0745	0.5744	0.2766	0.4401	6.0000

Supplementary Table 8. Fractional atomic coordinates for dynaCOF-330III (dioxane).

dynaCOF-330III (dioxane)<br>Space group: $P4_2/n$ (No.86); $a = b = 24.96$ Å; $c = 8.00$ Å;<br>$\alpha = \beta = \gamma = 90°$; $V = 4977$ Å^3; Z = 8					
**Atom**	**x**	**y**	**z**	**Occupancy**	B_{iso} **(Å^2)**
C1	0.0000	0.0000	0.5000	1.0000	5.0000
C10	0.8297	0.1654	0.7559	1.0000	10.0000
C11	0.7916	0.1858	0.6507	1.0000	10.0000
C12	0.8115	0.2032	0.5078	1.0000	10.0000
C13	0.8982	0.1828	0.5794	1.0000	10.0000
C14	0.8626	0.1944	0.4594	1.0000	10.0000
C15	0.7803	0.2346	0.3932	1.0000	3.6528
C16	0.2599	0.7975	0.3426	1.0000	3.6528
C17	0.7097	0.2137	0.2016	1.0000	3.6528
C18	0.6660	0.1809	0.1840	1.0000	3.6528
C19	0.6538	0.1371	0.2775	1.0000	3.6528
C2	0.0146	0.9517	0.3826	1.0000	10.0000
C20	0.3125	0.8748	0.4008	1.0000	3.6528
C21	0.2718	0.8412	0.4288	1.0000	3.6528
C22	0.7113	0.0591	0.5716	1.0000	3.6528
C23	0.9223	0.3339	0.3230	1.0000	3.6528
C24	0.0050	-0.7392	-0.3705	0.7500	2.0000
C25	0.0502	-0.7791	-0.3289	0.7500	2.0000
C26	0.0195	-0.7844	-0.0407	0.7500	2.0000
C27	-0.0260	-0.7446	-0.0788	0.7500	2.0000
C28	-0.9795	0.5705	0.0282	0.7500	2.0000
C29	-0.9478	0.6203	0.9722	0.7500	2.0000
C3	0.0560	0.9178	0.3929	1.0000	10.0000
C30	-0.9471	0.5880	0.6853	0.7500	2.0000
C31	-0.9788	0.5378	0.7378	0.7500	2.0000
C4	0.0628	0.8796	0.2773	1.0000	10.0000
C5	0.0358	0.8732	0.1361	1.0000	10.0000
C6	-0.0036	0.9079	0.1212	1.0000	10.0000
C7	-0.0085	0.9495	0.2388	1.0000	10.0000
C8	0.9183	0.1475	0.8459	1.0000	10.0000
C9	0.3820	0.6642	0.7733	1.0000	10.0000
H10	0.8128	0.1615	0.8785	1.0000	1.0000
H11	0.6954	0.7489	0.2031	1.0000	1.0000
H13	0.9391	0.1764	0.5369	1.0000	1.0000
H14	0.7058	0.6200	-0.1675	1.0000	1.0000

H18	0.8602	0.3089	0.4165	1.0000	1.0000
H21	0.2460	0.8506	0.5507	1.0000	1.0000
H22a	0.7526	0.0486	0.5314	1.0000	1.0000
H22b	0.6879	0.0225	0.6374	1.0000	1.0000
H22c	0.7099	0.0970	0.6579	1.0000	1.0000
H23a	0.5366	0.1496	0.2175	1.0000	1.0000
H23b	0.5738	0.1901	0.0544	1.0000	1.0000
H23c	0.6003	0.1917	0.2751	1.0000	1.0000
H24a	0.0186	-0.7111	-0.4745	0.7500	1.0000
H24b	-0.0321	-0.7621	-0.4128	0.7500	1.0000
H25a	0.0872	-0.7562	-0.2859	0.7500	1.0000
H25b	0.0603	-0.8038	-0.4446	0.7500	1.0000
H26a	0.0066	-0.8124	0.0645	0.7500	1.0000
H26b	0.0568	-0.7614	-0.0007	0.7500	1.0000
H27a	-0.0359	-0.7207	0.0387	0.7500	1.0000
H27b	-0.0632	-0.7674	-0.1218	0.7500	1.0000
H28a	-0.0059	0.5812	0.1397	0.7500	1.0000
H28b	-0.9505	0.5373	0.0651	0.7500	1.0000
H29a	-0.9222	0.6353	0.0806	0.7500	1.0000
H29b	-0.9770	0.6531	0.9326	0.7500	1.0000
H3	0.0829	0.9174	0.5045	1.0000	1.0000
H30a	-0.9762	0.6212	0.6485	0.7500	1.0000
H30b	-0.9201	0.5780	0.5745	0.7500	1.0000
H31a	-0.0047	0.5236	0.6291	0.7500	1.0000
H31b	-0.9498	0.5047	0.7753	0.7500	1.0000
H4	0.0961	0.8570	0.2918	1.0000	1.0000
H6	0.9690	0.9076	1.0018	1.0000	1.0000
H7	0.9593	0.9798	0.2259	1.0000	1.0000
H8	0.9441	0.1231	0.7838	1.0000	1.0000
N1	0.0587	0.8401	0.9953	1.0000	10.0000
O1	0.8816	0.3780	0.3308	1.0000	3.6528
O2	0.3222	0.9239	0.4371	1.0000	3.6528
O3	0.0315	-0.8159	-0.1906	0.7500	2.0000
O4	-0.0081	-0.7070	-0.2158	0.7500	2.0000
O5	-0.0142	0.5517	0.8849	0.7500	2.0000
O6	-0.9132	0.6064	0.8309	0.7500	2.0000

Supplementary Table 9. Fractional atomic coordinates for dynaCOF-330IV (dioxane).

dynaCOF-330IV (dioxane)<br>Space group: $P4_2/n$ (No.86); a = b = 27.84 Å; c = 7.26 Å;<br>$\alpha = \beta = \gamma = 90^\circ$; V = 5624.13 Å^3; Z = 8					
**Atom**	***x***	***y***	***z***	**Occupancy**	B_{iso} **(Å^2)**
C1	0.0000	0.0000	0.5000	1.0000	1.0000
C10	0.8400	0.1997	0.8091	1.0000	1.0000
C11	0.8691	0.1507	0.5649	1.0000	1.0000
C12	0.8379	0.1731	0.4446	1.0000	1.0000
C13	0.8098	0.2228	0.6876	1.0000	1.0000
C14	0.8060	0.2066	0.5088	1.0000	1.0000
C15	0.7780	0.2294	0.3907	1.0000	9.9949
C16	0.7307	0.2152	0.3643	1.0000	9.9949
C17	0.7020	0.2373	0.2265	1.0000	9.9949
C18	0.6535	0.2193	0.1984	1.0000	9.9949
C19	0.6364	0.1870	0.3047	1.0000	9.9949
C2	0.0239	0.9658	0.3699	1.0000	1.0000
C20	0.9480	0.3037	0.2979	1.0000	9.9949
C21	0.6650	0.1645	0.4405	1.0000	9.9949
C22	0.7107	0.1784	0.4701	1.0000	9.9949
C23	0.6808	0.0894	0.6538	1.0000	9.9949
C24	-0.2018	0.9935	-0.5848	1.0000	10.0000
C25	-0.2084	0.0142	-0.9091	1.0000	10.0000
C26	-0.2215	0.0663	-0.8585	1.0000	10.0000
C27	-0.2148	0.0454	-0.5302	1.0000	10.0000
C28	-0.9556	-0.3779	-0.3126	1.0000	9.9945
C29	-0.9866	-0.4176	-0.4002	1.0000	9.9945
C3	0.0046	0.9279	0.2748	1.0000	1.0000
C30	-0.0462	-0.4085	-0.1581	1.0000	9.9945
C31	-0.0159	-0.3687	-0.0677	1.0000	9.9945
C4	0.0692	0.9804	0.3405	1.0000	1.0000
C5	0.0974	0.9571	0.2108	1.0000	1.0000
C6	0.0320	0.9053	0.1417	1.0000	1.0000
C7	0.0789	0.9193	0.1112	1.0000	1.0000
C8	0.9054	0.1431	0.8700	1.0000	1.0000
C9	0.8712	0.1651	0.7452	1.0000	1.0000
H10	0.8414	0.2112	0.9569	1.0000	5.0000
H11	0.8948	0.1231	0.5124	1.0000	5.0000
H12	0.8365	0.1615	0.2969	1.0000	5.0000

H13	0.7835	0.2499	0.7407	1.0000	5.0000
H18	0.8702	0.2631	0.3933	1.0000	5.0000
H20a	0.5242	0.1716	0.1426	1.0000	5.0000
H20b	0.5676	0.2190	0.0896	1.0000	5.0000
H20c	0.5351	0.2199	0.3087	1.0000	5.0000
H22	0.7335	0.1607	0.5783	1.0000	5.0000
H23a	0.7060	0.1112	0.7404	1.0000	5.0000
H23b	0.7019	0.0672	0.5533	1.0000	5.0000
H23c	0.6592	0.0650	0.7448	1.0000	5.0000
H24a	-0.2141	0.9678	-0.4719	1.0000	5.0000
H24b	-0.1612	0.9904	-0.6029	1.0000	5.0000
H25a	-0.1678	0.0108	-0.9241	1.0000	5.0000
H25b	-0.2265	0.0039	-0.0445	1.0000	5.0000
H26a	-0.2085	0.0916	-0.9713	1.0000	5.0000
H26b	-0.2621	0.0696	-0.8428	1.0000	5.0000
H27a	-0.1967	0.0548	-0.3939	1.0000	5.0000
H27b	-0.2554	0.0488	-0.5151	1.0000	5.0000
H28a	-0.9160	-0.3850	-0.3433	1.0000	5.0000
H28b	-0.9664	-0.3415	-0.3716	1.0000	5.0000
H29a	-0.9759	-0.4540	-0.3406	1.0000	5.0000
H29b	-0.9811	-0.4179	-0.5558	1.0000	5.0000
H3	0.9658	0.9173	0.2974	1.0000	5.0000
H30a	-0.0858	-0.4021	-0.1267	1.0000	5.0000
H30b	-0.0348	-0.4449	-0.1008	1.0000	5.0000
H31a	-0.0222	-0.3688	-0.9125	1.0000	5.0000
H31b	-0.0265	-0.3322	-0.1269	1.0000	5.0000
H4	0.0833	1.0090	0.4237	1.0000	5.0000
H5	0.1341	0.9680	0.1973	1.0000	5.0000
H6	1.0168	0.8731	1.0721	1.0000	5.0000
H8	0.9441	0.1539	0.8652	1.0000	5.0000
N1	0.1038	0.8994	0.9568	1.0000	1.0000
O1	0.9088	0.3282	0.2033	1.0000	9.9949
O2	0.6455	0.1245	0.5437	1.0000	9.9949
O3	-0.2257	0.9811	-0.7580	1.0000	10.0000
O4	-0.1974	0.0792	-0.6786	1.0000	10.0000
O5	-0.9635	-0.3780	-0.1067	1.0000	9.9945
O6	-0.0388	-0.4078	-0.3577	1.0000	9.9945

Supplementary References

1. Goossens, R., Smet, M. & Dehaen, W. Bronsted- and Lewis acid-catalyzed cyclization giving rise to substituted anthracenes and acridines. *Tetrahedron Lett.* **43**, 6605-6608 (2002).

2. Oszlanyi, G. & Suto, A. The charge flipping algorithm. *Acta Cryst.*, A64, 123-134, (2008).

3. Sun, T., Wei, L., Chen, Y., Ma, Y. & Zhang, Y. B. Atomic-Level Characterization of Dynamics of a 3D Covalent Organic Framework by Cryo-Electron Diffraction Tomography. *J. Am. Chem. Soc.* **141**, 10962-10966 (2019).

4. Uribe-Romo, F. J. et al. A Crystalline Imine-Linked 3-D Porous Covalent Organic Framework. *J. Am. Chem. Soc.* **131**, (2009).

5. Yang, T. Y. et al. Yang, T. Y. et al.. Introduction of the X-Ray Diffraction Beamline of SSRF. *Nucl. Sci. Technol.* 26, 020101 (2015).

6. Gao, M. et al. Facile usage of a MYTHEN 1K with a Huber 5021 diffractometer and angular calibration inoperandoexperiments. *J. Appl. Crystallogr.* **49**, 1182-1189 (2016).

7. Material Studio 2017 (Accelrys Software Inc., San Diego, USA)

8. Plimpton, S. Fast Parallel Algorithms for Short-Range Molecular Dynamics. *J. Comput. Phys.* **117**, 1-19 (1995).

9. Kaminski, G. A., Friesner, R. A., Tirado-Rives, J. & Jorgensen, W. L. Evaluation and Reparametrization of the OPLS-AA Force Field for Proteins via Comparison with Accurate Quantum Chemical Calculations on Peptides. The *J. Phys. Chem. B* **105**, 6474-6487 (2001).

10. Dodda, L. S., Cabeza de Vaca, I., Tirado-Rives, J. & Jorgensen, W. L. LigParGen web server: an automatic OPLS-AA parameter generator for organic ligands. *Nucleic Acids Res.* **45**, W331-W336 (2017).

11. Verlet, L. Computer "Experiments" on Classical Fluids. II. Equilibrium Correlation Functions. *Phys. Rev.* **165**, 201-214 (1968).

12. Hoover, W. G. Canonical dynamics: Equilibrium phase-space distributions. *Phys. Rev. A* **31**, 1695-1697 (1985).
