# Supplementary material for: Guest-adaptive molecular sensing in a dynamic 3D covalent organic framework
Source: Nat Commun. 2022 Dec 24;13:7936. doi: 10.1038/s41467-022-35674-8 (PMC9790004; doi:10.1038/s41467-022-35674-8)
Supplement: Supplementary file 3 — Description of Additional Supplementary Files [file 41467_2022_35674_MOESM3_ESM.pdf]

Description of Additional Supplementary Files

File Name: Supplementary Data 1
Description: CIF of dynaCOF-330

File Name: Supplementary Data 2
Description: CIF of dynaCOF-330Ⅲ (acetone)

File Name: Supplementary Data 3
Description: CIF of dynaCOF-330Ⅳ (acetone)

File Name: Supplementary Data 4
Description: CIF of dynaCOF-330 Ⅰ (dioxane)

File Name: Supplementary Data 5
Description: CIF of dynaCOF-330Ⅲ (dioxane)

File Name: Supplementary Data 6
Description: CIF of dynaCOF-330Ⅳ (dioxane)
